# The Liability Threshold Model for Predicting the Risk of Cardiovascular Disease in Patients with Type 2 Diabetes: A Multi-Cohort Study of Korean Adults

**DOI:** 10.3390/metabo11010006

**Published:** 2020-12-24

**Authors:** Eun Pyo Hong, Seong Gu Heo, Ji Wan Park

**Affiliations:** 1 Molecular Neurogenetics Unit, Center for Genomic Medicine, Massachusetts General Hospital, Boston, MA 02114, USA; ehong5@mgh.harvard.edu; 2 Department of Neurology, Harvard Medical School, Boston, MA 02115, USA; 3 Medical and Population Genetics Program, the Broad Institute of M.I.T. and Harvard, Cambridge, MA 02142, USA; 4 Yonsei Cancer Institute, College of Medicine, Yonsei University, Seoul 03722, Korea; lukehur1@yuhs.ac; 5 Department of Medical Genetics, College of Medicine, Hallym University, Chuncheon, Gangwon-do 24252, Korea

**Keywords:** diabetic cardiovascular disease, functional gene network, genetic risk prediction, liability threshold model, polygenic risk score, population-based cohort study

## Abstract

Personalized risk prediction for diabetic cardiovascular disease (DCVD) is at the core of precision medicine in type 2 diabetes (T2D). We first identified three marker sets consisting of 15, 47, and 231 tagging single nucleotide polymorphisms (tSNPs) associated with DCVD using a linear mixed model in 2378 T2D patients obtained from four population-based Korean cohorts. Using the genetic variants with even modest effects on phenotypic variance, we observed improved risk stratification accuracy beyond traditional risk factors (AUC, 0.63 to 0.97). With a cutoff point of 0.21, the discrete genetic liability threshold model consisting of 231 SNPs (GLT_231_) correctly classified 87.7% of 2378 T2D patients as high or low risk of DCVD. For the same set of SNP markers, the GLT and polygenic risk score (PRS) models showed similar predictive performance, and we observed consistency between the GLT and PRS models in that the model based on a larger number of SNP markers showed much-improved predictability. In silico gene expression analysis, additional information was provided on the functional role of the genes identified in this study. In particular, *HDAC4*, *CDKN2B*, *CELSR2*, and *MRAS* appear to be major hubs in the functional gene network for DCVD. The proposed risk prediction approach based on the liability threshold model may help identify T2D patients at high CVD risk in East Asian populations with further external validations.

## 1. Introduction

Type 2 diabetes (T2D) keeps steadily increasing in prevalence in developed countries, and thus its complications, such as cardiovascular and renal diseases, constitute the leading cause of disease burden worldwide. The mortality rate in T2D patients with cardiovascular disease (CVD) is two to four times higher than in those with T2D only. In the United States, the majority of elderly patients with T2D die from heart disease (68%) and stroke (15%), even when their glucose levels are well controlled. CVD encompasses a broad spectrum of subphenotypes affecting the heart and blood vessels, including coronary artery disease (CAD), cerebrovascular disease (CVA), and peripheral arterial disease (PAD) [1]. The prevalence of diabetes among Korean adults aged 30 years or more increased from 12.4% in 2011 to 14.4% in 2016, and the highest prevalence estimate was seen in older adults aged 65 and over (i.e., 29.8%) [2]. Likewise, the prevalence of macrovascular complications in T2D patients estimated in 2011, such as CAD (10.3%), CVA (6.7%), and PAD (0.19%), is expected to increase further as the T2D prevalence increases in South Korea [3].

During the last decade, genome-wide association studies (GWAS) based on the “common disease common variant hypothesis” have successfully identified approximately 153 variants mapping to more than 120 T2D loci, including *PPARG*, *KCNJ11*, and *TCF7L2*, in multiethnic populations [4]. Although there is some overlap in susceptibility genes, previous studies have reported differences in genetic factors associated with CVD risk between diabetic and nondiabetic individuals. Multiple genes, such as *CDKN2A/2B*, *HNF1A*, *PCSK9*, *CELSR2-PSRC1-SORT1*, and *PHACTR1*, have been suggested to be associated with diabetic cardiovascular disease (DCVD) [5]. However, only one single-nucleotide polymorphism (SNP) of the *GLUL* gene, rs10911021, passed a threshold of genome-wide statistical significance for coronary heart disease (CHD) in non-Hispanic Caucasian patients with T2D (OR = 1.36, *p* = 2 × 10^−8^), and such a significant association was not observed in nondiabetic individuals [6].

To date, numerous statistical methods have been proposed to dissect the genetic architecture of complex traits. In particular, the use of a linear mixed model (LMM) in GWAS improves the statistical power to detect genetic associations by removing redundant SNPs [7]. Polygenic risk scoring (PRS) improves disease risk predictability by estimating the cumulative effect of multiple susceptibility variants [8]. However, a complex model that combines conventional risk factors, such as hypertension, obesity, and smoking, with a polygenic model may further enhance the predictive power for CVD risk in diabetic patients [9]. Another useful method for disease prediction, the liability threshold (LT) model, also called a probit model, gave the highest predictive accuracy compared to both the Risch risk model and the logit model using the same dataset. Here, liability refers to an individual’s innate tendency to develop a disease determined by the combinatory effects of genetic and environmental factors on the disease incidence [10]. While recent meta-analyses of GWAS have discovered many new T2D loci by increasing sample size, large-scale sequencing studies, contrary to expectations, have identified very few rare variants despite having sufficient statistical power [11].

The development of reliable prediction models for complex diseases, such as DCVD, is of the utmost importance in the era of precision medicine. To the best of our knowledge, risk prediction based on a multifactorial liability threshold model (MLT) that combines the effects of multiple genes and conventional nongenetic factors has not been applied to DCVD yet. In this study, we initially constructed genetic LT models with three different sets of DCVD-associated variants using data obtained from four Korean population-based cohort studies. Subsequently, we compared the discriminatory performance of three polygenic LT models for cardiovascular risk stratification in diabetic patients with the corresponding PRS models. In addition, we evaluated the degree of improvement in predictive performance for DCVD risk classification by adding genetic risk information to a phenotype-based risk model.

## 2. Results

### 2.1. Nongenetic Risk Factors for DCVD

Of the 21 nongenetic variables tested in this study, age was the most significant risk factor for DCVD (Table 1). Compared to T2D patients under the age of 50, the risk of developing CVD increased significantly in the 50s and 60s (OR = 2.28 and 3.75, *p* = 0.007 and 5.1×10^−6^, respectively) (Table S1). The mean serum creatinine level in the DCVD patient group (1.01 mg/dL) was significantly higher than that of the T2D control group (0.91 mg/dL) (OR = 2.62, *p* = 5.3 × 10^−5^). The effect of systolic blood pressure (SBP) on DCVD (OR = 1.01, *p* = 0.032) turned out to be statistically insignificant in multivariate analysis, whether treated as a continuous variable or as a categorical variable. In addition, past alcohol and tobacco consumption (OR = 1.96 and 1.43, *p* = 0.005 and 0.062, respectively), higher income (OR = 0.69, *p* = 0.027), total cholesterol (TC), triglycerides (TG), and gamma-glutamyl transpeptidase (GGT) were associated with DCVD risk in univariate analysis. However, only three variables, age (OR = 1.06, *p* = 6.5 × 10^−6^), BMI (OR = 1.09, *p* = 0.005), and blood creatinine level (OR = 2.02, *p* = 0.028), remained in the multivariate logistic regression (MLR) model after backward stepwise elimination.

### 2.2. Genetic Risk Factors for DCVD

In the current LMM analysis, after adjusting for age, sex, BMI, and creatinine level, two SNPs, rs4538911 (*LOC392180-MCPH1*, 8p23.2) and rs9982069 (*PPIAL3-SLC6A6P*, 21q21.1), showed the most significant associations with DCVD (*p* = 5.0 × 10^−7^ and 9.1 × 10^−7^, respectively) (Table 2). The regional association plots showed additional SNPs that were not in high LD (*r^2^* < 0.8) but yielded suggestive associations with DCVD (*p* < 0.05) in the vicinity of those SNPs (Figure S1).

Among the 169 genotyped tSNPs that were also previously reported to be associated with CVD and/or DCVD (*r^2^* < 0.8), 15 tSNPs yielded replicated associations with CVD in Korean T2D patients (0.001 < *p* < 0.05) (Table 2). The detailed LMM analysis results for 32 SNPs (*p* < 1 × 10^−4^) and 216 SNPs (*p* < 1 × 10^−3^) are provided in Table 2 and Table S2, respectively.

### 2.3. Gene Function Prediction

After filtering out 14 genes that did not appear in the DAVID database from 200 genes harboring 231 SNPs, we identified 92 significantly enriched GO terms associated with 118 genes (*p* < 0.05 and *FDR* < 0.1, data not shown). The most enriched GO term, GO:0007399~nervous system development, was associated with 45 genes, including *HDAC4*, *FGF9*, and *EPHA5* (*p* = 8.2 × 10^−8^, *FDR* = 1.5 × 10^−4^). Five genes, *EPHB2*, *EPHA3*, *EPHA5*, *EFNA5*, and *SLIT3*, were significantly enriched in axon guidance in the KEGG pathway, essential for neuronal network formation (hsa04360, *p* = 0.03, data not shown).

Of the 41 genes harboring 47 SNPs, four genes did not appear in DAVID. We identified 10 GO terms significantly enriched in 19 genes, and three of which, *HDAC4*, *NOX4*, and *NRP1*, were shown to play an important role in smooth muscle cell migration (GO:0014911, *p* = 0.001, *FDR* = 0.02) (Table 3).

**Figure 1:**
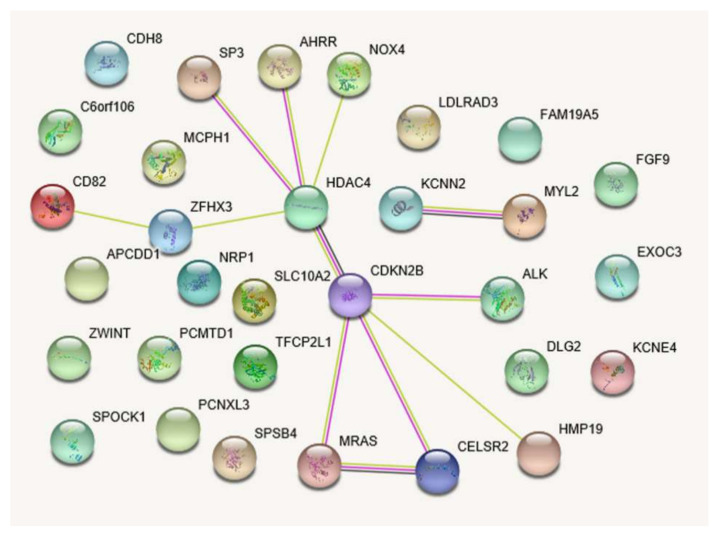
Protein–protein interaction network of 31 candidate genes for diabetic cardiovascular disease: Light-green line indicates the presence of co-publications found through text mining; light purple, evidence of homology; purple line, experimental evidence of coexpression; black line, evidence of mRNA coexpression (confidence score of STRING, 0.25).

Three other genes, *MYL2*, *FGF9*, and *MRAS*, were shown to be involved in the KEGG pathway hsa04810~regulation of actin cytoskeleton (*p* = 0.042, Figure S2). In particular, genes such as *HDAC4*, *CDKN2B*, *CELSR2*, and *MRAS* are major hubs in both functional networks for 31 and 170 genes that harbor 47 (*p* < 1 × 10^−4^) and 231 SNP sets (*p* < 1 × 10^−3^), respectively (Figure 1 and Figure S3).

### 2.4. DCVD Risk Prediction

#### 2.4.1. Genetic Risk Prediction

The disease-free mortality of Koreans aged 40 to 69 was higher in men than in women (19.5 vs. 7.3 per 10,000 people). However, the incidence rate (IR) of DCVD in T2D patients was higher in women than in men (15.67 vs. 13.47 per 1000 person-years) (Table S3). A model consisting of 15 previous SNPs that also showed nominally significant associations (*p* < 0.05) in this Korean study did not achieve sufficient predictability for DCVD (AUC, 53.7%). On the other hand, by adding SNPs that were less significantly associated with DCVD, the genetic liability threshold (GLT) model showed significantly improved predictability than the model using a more stringent *p*-value threshold for SNP selection (AUCs: 73.2% and 99.2% for GLT_47_ and GLT_231_, respectively). As the number of SNPs included in the model increased, the mean difference (MD) in liability to DCVD between cases and controls increased (MDs: 0.006, 0.044, and 0.216 for GLT_15_, GLT_47_, and GLT_231_, respectively). For every 1-point increase in normalized genetic liability on a scale of 0 to 10, the risk of developing DCVD also increased accordingly (ORs: 1.05, 1.54, and 14.13 for GLT_15_, GLT_47_, and GLT_231_, respectively) (Table 4).

When comparing the GLT and PRS model performance to predict DCVD risk in T2D patients, PRS_47_ performed better than GLT_47_ (ΔAUC = 11%); however, there was no significant difference between the two methods when predicting genetic risk based on a set of 15 or 231 SNP markers. In particular, we observed consistency between two risk measurements in that the model based on a larger number of SNP markers showed much-improved predictability (AUCs: 99.21% and 99.18% for GLT_231_ and PRS_231_, respectively) (Table 4). When we assigned each participant a percentile based on the GLT_231_ or PRS_231_ value, all DCVD patients have liability or risk scores above the 90th percentile in the risk distribution (Figure S4).

We further evaluated the predictive ability of discrete models to identify T2D patients at high CVD risk and found that GLT_231_ outperformed PRS_231_. Using a cutoff point of 0.21 or greater, the GLT_231_ model correctly classified 87.7% of 2378 individuals as high or low risk for DCVD with high sensitivity and specificity of 100% and 86.8%, respectively. Since there were no DCVD cases in the low-risk group, we could not estimate the ORs in the discrete GLT and PRS models (Table 4). When we stratified the liability and genetic risk scores into four risk quartiles and compared the highest (Q4) to the lowest quartile (Q1), the OR of each was large, possibly due to the small number of cases in the first quartile (OR = 20.2 and *p* = 6.3 × 10^−9^ for GLT_47_; OR = 30.5 and *p* = 1.2 × 10^−13^ for PRS_47_) (Table S4).

#### 2.4.2. Multifactorial Risk Prediction

**Figure 2:**
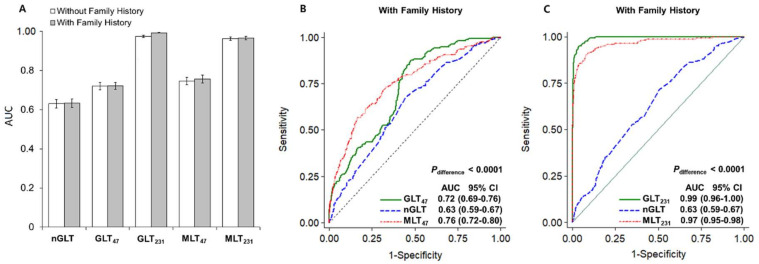
Comparison of the area under the ROC curves (AUCs) of three liability threshold (LT) models, nongenetic (nGLT), genetic (GLT), and multifactorial (MLT) models: (**A**) Bar graph with standard error bars for comparing AUC values of LT models with or without DCVD family history (grey-filled bars and transparent bars, respectively) (**B**,**C**). AUC statistics estimated for genetic (solid green lines), nongenetic (blue dashed lines), and multifactorial liability threshold (red tight-dotted lines) models, including a family history of DCVD for the 47- and 231-SNP sets, respectively.

We observed a much higher performance of risk stratification for CVD in T2D patients in the genetic models than in the nongenetic model (AUCs: 0.63 for nGLT vs. 0.99 for GLT_231_), whereas the GLT model, which includes a family history of DCVD, slightly improved the predictive performance (e.g., ΔAUC = 2% for GLT_231_). By adding four nongenetic risk factors, the predictability of the 47 SNP-based genetic models improved (ΔAUC = 4%), whereas the predictability of the 231 SNP-model slightly decreased (ΔAUC = −2%) (Figure 2). Specifically, the combined effect of the four nongenetic factors was weaker than that of the susceptibility SNPs (ORs: 1.23 for nGLT vs. 1.54 and 14.13 for GLT_47_ and GLT_231_, respectively), and these results were similar to those of the PRS models (ORs: 1.21 for nGRS vs. 2.72 and 18.41 for PRS_47_ and PRS_231_, respectively) (Table 4). As in the continuous model, the predictability for an individual’s DCVD risk increased in the quartile liability-based model by integrating nongenetic factors and 47 SNP information (ΔAUC = 2%). Contrary to expectations, the predictive performance of the PRS model was higher than that of the multifactorial model (ΔAUC = −11%) (Table S4).

We observed similar predictive performance in each of the four cohorts, although the case-control data from the Health2 Study showed the highest AUC values (Table S5). In 10-fold cross-validation, we also demonstrated consistency in the predictive performance of the models (Table S6). Since net reclassification improvement (NRI) has become a widely used measure to assess the predictive performance of risk models, we estimated the degree of improvement in continuous NRI achieved by adding genetic information to the nongenetic risk model. By adding 47 SNPs to the nGLT model, the enhanced model correctly assigned 12% of DCVD patients to higher predicted risk (event NRI, NRI_e_) and 32% of the control group to lower risk (non-event NRI, NRI_ne_). The overall NRI, calculated as the sum of NRI_e_ and NRI_ne_, was as large as 0.441, but the continuous NRI of the risk score-based model was greater than that of the liability-based model (NRI = 1.017 for PRS_47_). Compared to the adding effect of 47 SNP information in DCVD prediction, adding 231 SNP information improved the nongenetic model significantly (NRIs: 1.824 and 1.837 for GLT_231_ and PRS_231_, respectively) (Table S7).

## 3. Discussion

We validated the impact of traditional CVD risk factors, such as age, obesity, elevated blood pressure, cigarette smoking, and alcohol drinking, on the development of DCVD in Korean T2D patients [12]. Interestingly, a significant association between elevated serum creatinine, a clinical marker of renal dysfunction, and DCVD was observed in the case-control study, while the association with hypertension became more significant through the 10-year follow-up (data not shown). These results are consistent with the previous findings that diabetic nephropathy rarely occurred in patients with diabetes duration less than ten years and that diabetic patients with CVD complications were more likely to take antihypertensive drugs than those with T2D alone [13,14]. In this study, serum lipid or GGT levels were lower in the DCVD patient group than in the control group. Previous studies have reported that elevated GGT and CRP levels increased the risks of dyslipidemia, metabolic syndrome, and CVD, yet their prognostic values of CVD events in T2D patients remain controversial [15,16]. As reported in a general population-based cohort study, increased CVD risk due to low education and wealth levels has also been observed in Korean T2D patients [17].

In this LMM-based genetic association study, four SNPs, rs4538911, rs7946015, rs17465734, and rs9982069, did not achieve a genome-wide threshold of *p* < 5 × 10^−8^ but exhibited suggestive associations with DCVD risk (5 × 10^−7^ < *p* < 1 × 10^−5^). The SNP, rs10911021, located near the *GLUL* gene (1q25), which had been associated with CHD in Caucasian T2D patients, revealed no significant association in this East Asian study [6,18]. On the other hand, we found significant associations between DCVD and 15 reported SNPs located in or near CVD candidate genes, including *CELSR2-PSRC1*, *CDKN2B*, *TFCP2L1*, *HDAC4*, *MRAS*, *SPSB4*, *KCNN2*, *MYL2*, and *ZFHX3* (0.001 < *p* < 0.05) [5,19]. Whereas the minor G allele of rs599839, located 500 bp from the 3′-untranslated region (UTR) of the *PSRC1* gene (1p13.3), is a well-replicated variant in various subtypes of CVD and an intronic SNP, rs12801636, of the *PCNXL3* gene (11q13.1), is a validated SNP for lipid levels [20,21], others have not been implicated yet as trait-associated SNPs. However, the genes harboring intronic SNPs, *ALK* (rs4575680), *EPHA3* (rs1512909), and *TULP4*, also known as *TUSP* (rs341137), have been implicated in CVD-related traits, such as blood pressure, arterial fibrillation, and systemic sclerosis [22,23,24]. Genes near the intergenic SNPs identified in Korean patients with DCVD, especially the nearest gene to rs1401939 (2q22.1), *LINC01853*, a long intergenic noncoding RNA gene (lncRNA), was recently reported to be associated with coronary artery calcified atherosclerotic plaque in African-American T2D patients. Moreover, a nearby gene, *LRP1B*, a member of the LDL receptor gene family, has previously been implicated in CHD and heart failure [25]. The *ZWINT* gene neighboring rs1503908 has also been reported to be related to cardiac hypertrophy; however, the intergenic SNPs such as rs6750818, rs1154846, and rs9586032, have never been implicated in DCVD-related traits [26]. In silico functional analysis provides additional evidence to support the role of these genes in DCVD pathogenesis, particularly in the migration and proliferation of smooth muscle cells that occur after vascular damage. In particular, network analysis highlighted the hub genes in the PPI network, such as *CDKN2B*, *HDAC4*, *CELS2*, and *MYL2*.

Predicting individual disease risk is at the core of precision medicine to prevent disease progression in susceptible individuals through early intervention and lifestyle management. The GRS model, which combines a small number of susceptibility SNPs identified by GWAS, has been replaced by the PRS model that incorporates the effects of a larger number of SNPs passing a less stringent association *p*-value threshold to improve statistical power [27]. The PRS method has shown the potential to improve risk stratification accuracy beyond traditional risk factors [28]. According to a European study, the AUC of each GRS model for CHD prediction consisting of five SNPs, seven clinical predictors, or both GRS plus clinical predictors were 0.577, 0.699, and 0.715, respectively [19]. In a large-scale study of CAD risk prediction in T2D patients, adding a weighted GRS comprised of 204 CAD candidate SNPs to a model of 13 clinical predictors such as age, sex, history of CAD, smoking habits, and SBP lead to an 8% improvement in risk classification. However, the AUCs of the models did not appear to be good enough to distinguish high- and low-risk individuals (i.e., genetic 0.567, clinical 0.675, combined 0.681), and all participants were of European ancestry [29]. Recently, the issue of limited generalizability of European derived PRS has been raised, and the importance of developing PRS specific to non-European populations is emphasized [30,31].

In the current study, we constructed non-logit probit models, also called liability threshold models, to predict DCVD risk by combining effects of a set of 47- or 231-tSNPs selected according to the level of statistical significance and observed much-improved model performance in GLT_231_ compared to GLT_47_ (i.e., AUC, 0.99 vs. 0.73). By including 231 tSNPs and family history information, the predictability of the nongenetic model comprising age, sex, BMI, and blood creatinine level greatly improved in AUC from 0.63 to 0.97; however, the predictability of the genetic model was higher than that of the multifactorial model (ΔAUC = 2%). We found similar prediction estimates for DCVD risk in each of the four cohorts and validated the performance of these models in 10-fold cross-validation. These results were consistent with observations from discrete- and quantile-based analyses. Besides, we observed consistency between the two risk measures, liability- and risk score-based models, in that the model based on a larger number of SNP markers showed much-improved predictability (AUCs: 99.21% and 99.18% for GLT_231_ and PRS_231_, respectively).

Previous studies have raised concerns about the interpretation of the clinical significance of a small change in AUC and the tendency of NRI to make uninformative markers appear predictive [32,33]. Although we analyzed 2378 T2D patients obtained from the four largest population-based cohorts in Korea, 168 DCVD cases may not be enough to develop a risk model specific to CVD subtypes such as CAD. Moreover, the high AUC and NRI statistics observed in the GLT_231_ and PRS_231_ models might represent an overfitting issue that often occurs when analyzing a large number of SNP markers in a relatively small number of samples. However, CAD itself consists of heterogeneous subtypes, and the shared genetic factors may underlie the pervasive pleiotropy among CVD subtypes [34]. Furthermore, the lack of statistical significance does not necessarily preclude the presence of an association of a risk factor with the disease. Additional efforts are necessary to implement a risk prediction model in clinical practice, such as developing a set of genetic markers with excellent DCVD risk classification performance, improving the predictive performance of risk models, and validating the promising model in independent datasets.

## 4. Materials and Methods

### 4.1. Study Populations

To explore potential risk factors for DCVD, we first identified 2378 T2D patients from 16,147 participants with comparable genetic and clinical data collected from the initial surveys of four population-based Korean cohort studies established by the Center for Genome Science at the Korean National Institute of Health: the Korea Association Resource Study (KARE), Health Examinees (HEXA) Study, Korean Healthy Twin Study (HT), and Health2 Study. Based on the International Diabetes Federation guidelines (https://www.idf.org/), T2D cases were defined as fasting plasma glucose (FPG) ≥ 126 mg/dL, 2-h plasma glucose after 75 g oral glucose tolerance test (2 h OGTT) ≥ 200 mg/dL or with a medical history of T2D.

We identified 168 T2D patients with a medical history of myocardial infarction (MI), CAD, congestive heart failure (CHF), PAD, or CVA (mean age 61.1 ± 0.5 years) and 2210 T2D patients without any history of CVD (mean age 56.9 ± 0.2 years) at the baseline survey conducted from 2001 to 2002. All participants provided written informed consent, and details of each cohort are described elsewhere [35,36,37,38]. This study also obtained Institutional Review Board approval of Hallym University (HIRB-2014-109).

### 4.2. Genotyping and Quality Controls

Genomic DNA derived from the peripheral blood of participants was genotyped using Genome-wide Human SNP array 5.0 in the KARE study and SNP array 6.0 in the other three cohort studies (Affymetrix Inc., Santa Clara, CA, USA). We found 352,228, 516,610, 606,876, and 627,659 SNPs that passed the quality control filters (i.e., genotyping call rate ≥ 95%, minor allele frequency ≥ 1%, and Hardy-Weinberg equilibrium *p*-value ≥ 1 × 10^−6^) in the KARE, HEXA, Twin-family, and Health2 studies, respectively [35,36,37,38]. We computed linkage disequilibrium (LD), represented as *r^2^*, between SNP pairs using Haploview software [39]. To fill in both missing genotypes and untyped markers, we imputed genotypes at an additional > 4.4 million SNP loci using the East Asian reference panel of the 1000 Genomes Project with IMPUTE2 [40].

### 4.3. Statistical Analysis

#### 4.3.1. Association of Conventional Risk Factors with the Development of DCVD

To identify nongenetic risk factors associated with DCVD, we initially conducted univariate logistic regression analyses to estimate odds ratios (ORs) and 95% confidence intervals (CIs) for age, sex, family history of T2D or DCVD, four environmental, and thirteen clinical variables by comparing 168 DCVD patients with 2210 T2D patients at baseline. We then selected a set of informative covariates by a backward elimination procedure (Table 1). All analyses were performed using STATA software package v.11.2 (Stata Corp., College Station, TX, USA).

#### 4.3.2. Genetic Association Analysis of DCVD Based on Generalized Linear Mixed Model

We initially performed genome-wide GLMM analysis under an additive genetic model after adjusting for age, sex, BMI, and creatinine level using 210,830 autosomal tagging SNPs (tSNPs) after removing redundant SNPs (*r^2^* > 0.8) in 168 DCVD cases and 2210 T2D controls as implemented in Genome-wide Complex Trait Analysis (GCTA) v1.24 [41]. We generated Manhattan plots using the R package ‘qqman’ (https://cran.r-project.org/web/packages/qqman) and further explored the ±500-kb regions adjacent to the significant SNPs using a web-based program, LocusZoom v1.3 (http://locuszoom.org/).

We also identified the SNPs associated with CVD or DCVD by searching for review articles in PubMed and web databases, such as GWAS Catalog (https://www.ebi.ac.uk/gwas/) and HuGe Navigator (https://phgkb.cdc.gov/HuGENavigator/home.do), until 6 September 2018. For 231 SNPs, including 216 SNPs identified here at *p* < 1 × 10^−3^ plus 15 reported SNPs replicated at *p* < 0.05 in the present study, we conducted GLMM analyses using STATA after adjusting for the four covariates shown above.

#### 4.3.3. Gene Functional Enrichment, Pathway, and Network Analyses

We analyzed the enrichment of gene ontology (GO) terms and the Kyoto Encyclopedia of Genes and Genomes (KEGG) pathways to group the candidate SNPs into functionally annotated gene sets using the web application of the Database for Annotation, Visualization and Integrated Discovery (DAVID) v6.8 [42]. Biological functions with a false discovery rate (FDR) < 10% were considered to be strongly enriched in the annotation categories. Furthermore, we displayed the protein–protein interaction (PPI) network for the selected gene list using STRING v11 [43].

#### 4.3.4. Risk Prediction of DCVD in T2D Patients Using

##### Incidence-Based Liability Threshold Models

The lifetime risk (i.e., incidence-based risk) of developing DCVD in an individual with T2D was estimated based on the measured liabilities, age- and sex-specific incidence rates (IRs), and disease-free mortalities obtained from the Korean Statistical Information System (KOSIS, www.kosis.kr) (Table S3).

The liability to DCVD was estimated based on the prevalence, additive relative risk (RR), heritability (*h^2^*), and family history of DCVD using the method proposed by So et al. (2011) [44] We applied the CVD prevalence among Korean adults with T2D (17%) and *h^2^* of 0.5 [3,45]. Genetic and nongenetic factors were categorized into 0, 1, or 2, and measurable liability for each individual was estimated from the equation $L=\sum_{i}\beta_{i}x_{i}+\sum_{j}\beta_{j}x_{j}+e$, where $x_{i}$ and $x_{j}$ denote the risk allele count at the *i*th susceptibility locus and the risk score of the *j*th nongenetic variable, respectively. The residual, *e*, represents the liability contributed by the unknown risk factors. The detailed procedure is described elsewhere [44,46]. Based on the individual lifetime risk to DCVD, we constructed genetic, nongenetic, and multifactorial LT models (i.e., GLT, nGLT, and MLT) for 47- and 231-SNP sets selected at *p* < 10^−4^ and *p* < 10^−3^, respectively. We further examined the predictive performance of discrete (L—low risk, H – high risk) and quartile models (Q1—lowest risk, Q2—low risk, Q3—high risk, Q4—highest risk) for DCVD risk prediction. We also compared the predictability of each risk model on DCVD observed in each of the four cohort studies and validated them using 10-fold cross-validation.

##### Polygenic Risk Scores

To compare the predictive performance for the DCVD risk with the GLT models, the PRS for each of the three SNP set, PRS_15_, PRS_47_, and PRS_231_, were constructed based on the formula, *PRS* = $\overset{m}{\underset{i=1}{\sum }}(logOR_{i}\times x_{i})$, where $x_{i}$ denotes the risk allele count at the *i*th susceptibility locus [34]. Two measures of risk, GLT and PRS, each with values in different ranges, were converted into a common scale of 0–10 using a formula for min-max normalization, $X_{N}=\left(\frac{X-X_{min}}{X_{range}}\right)\times 10$, where *X_N_* is the normalized values, *X* is the original values, *X_min_* is the minimum value on the original scale, and *X_range_* is the difference between the maximum score and the minimum score on the original scale [47]. We compared the predictability of the GLT models with the PRS models based on the interpretation of the AUC and continuous NRI using the STATA commands, ‘reccomp’ and ‘incrisk’, respectively [48]. All analyses were conducted using two statistical software packages, Stata and R.

## 5. Conclusions

We validated the impact of traditional CVD risk factors such as age, obesity, elevated blood pressure, cigarette smoking, and alcohol drinking on the development of DCVD in Korean T2D patients. We also replicated significant associations with DCVD for 15 previously reported SNPs located in or near CVD candidate genes. In silico gene expression analysis lent further support to the functional roles of these genes in DCVD pathogenesis, particularly in the migration and proliferation of smooth muscle cells that occur after vascular damage, and highlighted the hub genes in the PPI network, such as *CDKN2B*, *HDAC4*, *CELS2*, and *MYL2*. For the same set of SNP markers, the GLT and PRS models showed similar predictive performance. Using the genetic variants that have even modest effects on phenotypic variance, it is possible to improve risk stratification accuracy beyond traditional risk factors. In conclusion, the polygenic LT model developed in an ethnically homogenous Korean population may help identify T2D patients at high risk of CVD in East Asians genetically similar to Koreans.

## Figures and Tables

**Table 1:** Risk of cardiovascular disease in Korean patients with type 2 diabetes according to environmental and clinical characteristics.

	DCVD	T2D Only	Logistic Regression ^†^	
Characteristics *	(N = 168)	(N = 2210)	OR (95% CI)	*p*
Men, N (%)	91 (54.2)	1159 (52.4)	1.07 (0.78–1.47)	0.666
Age, years (%)	61.1 ± 0.5	56.9 ± 0.2	1.07 (1.05–1.09)	3.2 × 10^−9 ‡^
Income, N (%)				
<1 million won	82 (48.8)	908 (41.1)	Reference	
1 million won ≤	69 (41.1)	1110 (50.2)	0.69 (0.49–0.96)	0.027
Education, N (%)				
<High school	108 (64.3)	1444 (65.3)	Reference	
High school ≤	58 (34.5)	752 (34.0)	1.03 (0.74–1.44)	0.856
Smoking status, N (%)				
Nonsmoker	93 (55.4)	1255 (56.8)	Reference	
Current smoker	30 (17.9)	510 (23.1)	0.79 (0.52–1.21)	0.286
Ex-smoker	45 (26.8)	426 (19.3)	1.43 (0.98–2.07)	0.062
Drinking status, N (%)				
Nondrinker	75 (44.6)	1042 (47.2)	Reference	
Current drinker	65 (38.7)	973 (44.0)	0.93 (0.66–1.31)	0.670
Ex-drinker	26 (15.5)	184 (8.3)	1.96 (1.22–3.15)	0.005
Family history, N (%)				
T2D, yes	112 (66.7)	1367 (61.9)	0.96 (0.66–1.39)	0.824
DCVD, yes	13 (11.7)	82 (5.5)	1.22 (0.66–2.25)	0.530
BMI, kg/m^2^	25.9 ± 0.3	25.2 ± 0.1	1.06 (1.01–1.12)	0.012 ^‡^
SBP, mm Hg	131.1 ± 1.5	127.9 ± 0.4	1.01 (1.00–1.02)	0.032
DBP, mm Hg	80.8 ± 0.9	79.8 ± 0.2	1.01 (0.99–1.02)	0.256
TC, mg/dL	192.9 ± 3.3	201.1 ± 0.9	0.995 (0.992–0.999)	0.018
TG, mg/dL	176.0 ± 7.5	199.7 ± 3.2	0.998 (0.997–0.999)	0.042
GGT, IU/L	38.4 ± 2.5	57.8 ± 2.7	0.996 (0.992–1.000)	0.027
AST, IU/L	29.8 ± 1.3	32.2 ± 0.6	0.995 (0.985–1.004)	0.246
ALT, IU/L	30.9 ± 1.5	33.7 ± 0.8	0.996 (0.988–1.003)	0.263
Creatinine, mg/dL	1.01 ± 0.03	0.91 ± 0.01	2.62 (1.64–4.19)	5.3 × 10^−5 ‡^
CRP, mg/L	2.73 ± 0.49	2.57 ± 0.10	1.01 (0.98–1.04)	0.689
FPG, mg/dL	124.7 ± 3.3	137.0 ± 1.2	0.99 (0.99–1.00)	0.004
2 h PG, mg/dL	200.3 ± 10.7	240.9 ± 3.0	0.99 (0.99–1.00)	0.001
Hemoglobin A1c	7.09 ± 1.49	7.44 ± 1.74	0.87 (0.74–1.03)	0.101

ALT, alanine transaminase; AST, aspartate aminotransferase; BMI, body mass index; CI, confidence interval; CRP, C-reactive protein; DBP, diastolic blood pressure; DCVD, diabetic cardiovascular disease; FPG, fasting plasma glucose; GGT, gamma-glutamyl transpeptidase; OR, odds ratio; SBP, systolic blood pressure; TC, total cholesterol; TG, triglyceride; T2D, type 2 diabetes mellitus; 2 h PG, 2-h plasma glucose after 75 g oral glucose tolerance test. * Data are shown as the number of subjects (percentage) for categorical variables and mean ± standard deviation for continuous variables. The mean values of 2 h PG and hemoglobin A1c were estimated from the KARE data. ^†^ ORs, 95% CIs, and *p*-values were estimated by comparing 168 DCVD cases to 2210 T2D controls selected from the initial surveys of four cohort studies using univariate logistic regression analysis. ^‡^ The variables remained statistically significant at *p* < 0.05 after backward stepwise selection in the multivariate logistic regression model.

**Table 2:** Results of linear mixed model analysis of 47 candidate SNPs for diabetic cardiovascular disease.

Gene	Chr	SNP	Function	N/R	LMM ^†^		
					RAF (Ca/Co)	OR	*p*
*15 previously reported SNPs (p < 0.05)* ^‡^							
*LOC107986441 (KCNN2*) *	5q22.2	rs4621553	intron	A/G	0.09/0.05	1.05	0.002
*MRAS* *	3q22.3	rs9818870	3’ UTR	C/T	0.03/0.01	1.08	0.011
*CELSR2, PSRC1* *	1p13.3	rs599839	500bp~3′ UTR	A/G	0.09/0.06	1.04	0.015
*IBTK* *	6q14.1	rs16893526	intergenic	G/A	0.15/0.11	1.03	0.017
*ZFHX3* *	16q22.3	rs879324	intron	A/G	0.67/0.62	1.02	0.022
*CDKN2B* *	9p21.3	rs1333042	intron	A/G	0.71/0.65	1.02	0.025
*MREGP1*	12p11.21	rs11610422	intergenic	A/G	0.07/0.05	1.04	0.027
*LOC100288146*	4q24	rs17035270	intron	C/T	0.99/0.04	1.04	0.028
*SPSB4* *	3q23	rs16851055	intron (ncRNA)	G/A	0.23/0.18	1.02	0.036
*ILRUN* (*C6orf106*) *	6p21.31	rs2814993	intron	G/A	0.03/0.01	1.07	0.037
*MTAP* *	9p21.3	rs7865618	intron	G/A	0.90/0.86	1.02	0.037
*PCNXL3*	11q13.1	rs12801636	intron	A/G	0.56/0.49	1.02	0.038
*HDAC4* *	2q37.3	rs6706785	intergenic	G/T	0.32/0.27	1.02	0.040
*TFCP2L1* *	2q14.2	rs17006292	intron	C/A	0.04/0.03	1.05	0.043
MYL2*	12q24.11	rs3782889	intron	G/A	0.88/0.83	1.02	0.046
*32 SNPs associated with DCVD (p < 10^−4^)*							
*MCPH1* *	8p23.2	rs4538911	intergenic	C/G	0.13/0.06	1.08	5.0 × 10^−7^
*LOC100505973*	21q21.1	rs9982069	intergenic	G/A	0.49/0.38	1.04	9.1 × 10^−7^
*CDH11* *	16q21	rs17465734	intergenic	T/A	0.05/0.01	1.14	8.0 × 10^−6^
*CD82* *	11p11.2	rs7946015	intergenic	A/T	0.26/0.17	1.04	8.2 × 10^−6^
*FAM19A5 (TAFA5)* *	22q13.31	rs5768165	intergenic	G/T	0.11/0.05	1.07	1.3 × 10^−5^
		rs2338258	intergenic	T/C	0.13/0.07	1.06	3.6 × 10^−5^
		rs5768143	intergenic	C/T	0.13/0.07	1.05	9.1 × 10^−5^
*MGC45800*	4q34.3	rs17072597	intron	C/T	0.22/0.14	1.05	1.5 × 10^−5^
*KCNE4* *	2q36.1	rs16864293	intergenic	T/A	0.09/0.04	1.08	1.6 × 10^−5^
*SLC9A3* *	5p15.33	rs1053226	intron	C/T	0.05/0.02	1.11	1.8 × 10^−5^
*SP3* *	2q31.1	rs41326844	intergenic	T/C	0.47/0.36	1.03	2.5 × 10^−5^
*AHRR* *	5p15.33	rs6555242	intron	T/G	0.07/0.03	1.09	3.1 × 10^−5^
*VAPA* *	18p11.22	rs16956185	intergenic	G/A	0.15/0.08	1.06	3.2 × 10^−5^
*ZWINT* *, *MIR3924*	10q21.1	rs1503908	intergenic	A/G	0.19/0.12	1.05	3.9 × 10^−5^
*NOX4* *	11q14.3	rs319025	intron	T/C	0.67/0.56	1.03	4.1 × 10^−5^
*SPOCK1* *	5q31.2	rs6893667	intergenic	C/T	0.06/0.02	1.10	4.2 × 10^−5^
*C14orf64 (LINCO1550)*	14q32.2	rs877455	intergenic	G/A	0.10/0.05	1.07	4.8 × 10^−5^
*LDLRAD3* *	11p13	rs1001715	intron	G/A	0.44/0.33	1.03	4.9 × 10^−5^
		rs12276510	intron	G/A	0.43/0.33	1.03	5.5 × 10^−5^
*ST18* *	8q11.23	rs2450153	intergenic	G/A	0.63/0.52	1.03	5.3 × 10^−5^
		rs3843918	intergenic	T/C	0.46/0.44	1.03	7.0 × 10^−5^
*CYP2B6* *	19q13.2	rs1872125	intron	T/C	0.24/0.16	1.04	5.7 × 10^−5^
*FGF9* *	13q12.11	rs9506827	intergenic	T/C	0.29/0.20	1.04	5.9 × 10^−5^
*MIRN656*	14q32.31	rs8016145	intergenic	G/A	0.09/0.04	1.08	6.4 × 10^−5^
*DLG2* *	11q14.1	rs349083	intron	G/A	0.47/0.36	1.03	6.5 × 10^−5^
*LOC646700*	9p21.1	rs10968749	intergenic	A/G	0.19/0.12	1.04	7.4 × 10^−5^
*METTL21EP, SLC10A2* *	13q33.1	rs9586032	intergenic	G/A	0.23/0.15	1.04	7.4 × 10^−5^
*PPIAL3*	21q21.1	rs2825256	intergenic	T/A	0.67/0.55	1.03	7.4 × 10^−5^
*HMP19* *	5q35.2	rs2913472	intergenic	A/C	0.05/0.02	1.11	7.9 × 10^−5^
*ALK* *	2p23.2	rs4575680	intron	G/C	0.08/0.04	1.07	9.0 × 10^−5^
*MIR1261*	11q14.3	rs10501726	intergenic	A/T	0.08/0.04	1.08	9.5 × 10^−5^
*NRP1* *	10p11.22	rs767164	intergenic	T/A	0.30/0.21	1.04	9.8 × 10^−5^

bp, base pair; Ca/Co, cases/controls; Chr., chromosome; LMM, linear mixed model; ncRNA, noncoding RNA; N/R, non-risk/risk allele; OR, odds ratio; RAF, risk allele frequency; SNP, single nucleotide polymorphism; UTR, untranslated region. * Genes linked to more than one Gene Ontology term. ^†^ The risk allele frequencies were estimated for cases (left) and controls (right). ORs and *p*-values were estimated in linear mixed models after adjusting for age, sex, body mass index, and serum creatinine level. ^‡^ 15 previously reported SNPs that were replicated in the current LMM analysis (*p* < 0.05).

**Table 3:** Gene Ontology functional enrichment analyses of 31 differentially expressed genes in diabetic cardiovascular disease.

Biological Function *	Gene, N	*p* ^†^	FDR, % ^†^	Gene Set
GO:0014911~positive regulation of smooth muscle cell migration	3	0.0012	2.0	*NOX4, HDAC4, NRP1*
GO:0048731~system development	16	0.0014	2.3	*NOX4, NRP1, MYL2, FGF9, MRAS, TFCP2L1, SPOCK1, CELSR2, ALK, APCDD1, HDAC4, CDKN2B, SP3, MCPH1, ZFHX3, DLG2*
GO:0061061~muscle structure development	6	0.0026	4.2	*NOX4, HDAC4, MYL2, FGF9, MRAS, ZFHX3*
GO:0048513~animal organ development	13	0.0027	4.3	*NOX4, NRP1, MYL2, FGF9, MRAS, TFCP2L1, CELSR2, APCDD1, HDAC4, CDKN2B, SP3, MCPH1, ZFHX3*
GO:0007517~muscle organ development	5	0.0030	4.8	*HDAC4, MYL2, FGF9, MRAS, ZFHX3*
GO:0014910~regulation of smooth muscle cell migration	3	0.0035	5.6	*NOX4, HDAC4, NRP1*
GO:0014909~smooth muscle cell migration	3	0.0040	6.4	*NOX4, HDAC4, NRP1*
GO:0048523~negative regulation of cellular process	15	0.0048	7.5	*NOX4, NRP1, MYL2, FGF9, TFCP2L1, SPOCK1, APCDD1, HDAC4, AHRR, CDKN2B, SP3, ZWINT, MCPH1, ZFHX3, DLG2*
GO:0007275~multicellular organism development	16	0.0055	8.7	*NOX4, NRP1, MYL2, FGF9, MRAS, TFCP2L1, SPOCK1, CELSR2, ALK, APCDD1, HDAC4, CDKN2B, SP3, MCPH1, ZFHX3, DLG2*
GO:0014812~muscle cell migration	3	0.0055	8.7	*NOX4, HDAC4, NRP1*

FDR, false discovery rate; GO, gene ontology * Categories of GO terms. ^†^ Fisher’s exact *p*-values and FDRs for each GO term were estimated using the DAVID tool.

**Table 4:** Comparison of predictive performance between genetic liability threshold model and polygenic risk score model for predicting diabetic cardiovascular disease in T2D patients.

Model	Ca/Co, N *	Ca/Co, Mean (Range) ^†^	OR (95% CI) ^‡^	*p*-Value	AUC
Nongenetic					
nGLT	167/2195	0.24 (0.06–0.40)/0.20 (0.06–0.40)	1.23 (1.15–1.32)	4.8 × 10^−9^	0.63 (0.59–0.67)
nGRS	167/2195	2.32 (0.00–4.25)/1.78 (0.00–4.41)	1.21 (1.13–1.29)	8.9 × 10^−9^	0.64 (0.60–0.68)
Genetic					
GLT_15_	164/2172	0.15 (0.11–0.23)/0.15 (0.10–0.26)	1.05 (0.99–1.10)	0.089	0.54 (0.49–0.58)
GLT_47_	163/2076	0.25 (0.14–0.42)/0.20 (0.12–0.39)	1.54 (1.41–1.68)	7.3 × 10^−22^	0.73 (0.70- 0.77)
GLT_231_	114/1558	0.38 (0.21–0.66)/0.16 (0.06–0.45)	14.13 (9.08–21.97)	7.4 × 10^−32^	0.99 (0.99–0.99)
L: < 0.21		0 (0.0)/1911 (86.5)	Reference	NA	0.93 (0.93–0.94)
H: 0.21 ≤		168 (100.0)/299 (13.5)	NA	NA	100/86.8/87.7 ^§^
PRS_15_	164/2172	0.27 (0.16–0.42)/0.26 (0.12–0.48)	1.16 (1.02–1.30)	0.019	0.55 (0.50–0.60)
PRS_47_	163/2076	0.93 (0.51–1.50)/0.69 (0.29–1.31)	2.72 (2.38–3.10)	3.0 × 10^−49^	0.84 (0.81–0.87)
PRS_231_	114/1558	5.40 (4.57–6.82)/4.19 (3.28–5.81)	18.41 (11.17–30.35)	3.2 × 10^−30^	0.99 (0.99–0.99)
L: < 4.57		0 (0.0)/ 1369 (62.0)	Reference	NA	0.81 (0.80–0.82)
H: 4.57 ≤		168 (100.0)/ 841 (38.1)	NA	NA	100/61.9/64.6 ^§^
Multifactorial					
MLT_47_	162/2062	0.23 (0.06–0.38)/0.17 (0.04–0.38)	1.84 (1.65–2.04)	1.9 × 10^−29^	0.76 (0.72–0.80)
MLT_231_	113/1552	0.41 (0.08–0.72)/0.15 (0.02–0.51)	7.79 (5.67–10.68)	4.9 × 10^−37^	0.97 (0.95–0.99)
MRS_47_	162/2062	3.26 (0.68–5.43)/2.47 (0.37–5.40)	1.39 (1.28–1.51)	2.5 × 10^−15^	0.71 (0.67–0.76)
MRS_231_	113/1552	7.71 (4.57–9.99)/5.92 (3.48–8.74)	2.98 (2.48–3.58)	3.0 × 10^−31^	0.86 (0.82–0.89)

AUC, area under the receiver operating characteristic curve; Ca/Co, Case/Control; CI, confidence interval; DCVD, diabetic cardiovascular disease; GLT, genetic liability threshold model; MLT, multifactorial liability threshold model; MRS, multifactorial risk score model; N, number; nGLT, nongenetic liability threshold model; nGRS, nongenetic risk score model; OR, odds ratio; PRS, polygenic risk score. * The number of cases and controls for each PRS model was the same as the GLT model based on the same number of SNPs. ^†^ Mean and range of liability or risk score groups using three sets of single nucleotide polymorphism markers (i.e., 15, 47, and 231 SNPs) for each of the case and control groups. For the discrete GLT_231_ and PRS_231_ models, the numbers and percentages of cases and controls were shown. ^‡^ ORs, 95% CIs, and *p*-values were estimated using logistic regression analysis for every 1-point increase in the standardized values of liability and polygenic risk score, respectively. ^§^ The AUCs of three liability threshold models were computed with a family history of DCVD. Sensitivity/Specificity/Percentage of persons correctly classified for DCVD status based on each categorical model.

## Data Availability

Restrictions apply to the availability of these data. Data was obtained from the National Biobank of Korea, the Center for Disease Control and Prevention (KCDC) and are available from https://is.cdc.go.kr/ with the permission of the KCDC.

## Supplementary Materials

The following are available online at https://www.mdpi.com/2218-1989/11/1/6/s1, Figure S1: Regional association plots for regions containing each of two SNPs, rs4538911 (LOC392180-MCPH1, 8p23.2) and rs9982069 (PPIAL3-SLC6A6P, 21q21.1), Figure S2: Schematic diagram of the regulation of actin cytoskeleton pathway (KEGG pathway, hsa04810), Figure S3: Protein-protein interaction network of 170 candidate genes for diabetic cardiovascular disease, Figure S4: The frequencies of cases and controls and their risks of DCVD by risk percentiles for genetic (GLT), nongenetic (nGLT), and multifactorial liability threshold (MLT) models of 168 DCVD cases and 2210 T2D controls, Table S1: Logistic regression analysis for association between diabetic cardiovascular disease and five nongenetic factors transformed into categorical variables, Table S2: Results of the linear mixed model analysis of 231 candidate SNPs for diabetic cardiovascular disease, Table S3: Incidence rates of cardiovascular disease in patients with type 2 diabetes and disease-free mortality rates in Korea, Table S4: Association results of risk prediction models for diabetic cardiovascular disease after stratification into risk quartiles in the case-control study, Table S5: Predictability of genetic and nongenetic liability threshold models on diabetic cardiovascular disease in KARE, HEXA, Health2, and Twin-family Studies, Table S6: Predictability of genetic and nongenetic liability threshold models on diabetic cardiovascular disease after 10-fold cross validation test, Table S7: Reclassification improvement achieved by adding genetic markers to the multifactorial models for diabetic cardiovascular disease.
