# Supplementary material for: The Liability Threshold Model for Predicting the Risk of Cardiovascular Disease in Patients with Type 2 Diabetes: A Multi-Cohort Study of Korean Adults
Source: Metabolites. 2020 Dec 24;11(1):6. doi: 10.3390/metabo11010006 (PMC7824099; doi:10.3390/metabo11010006)
Supplement: Supplementary file 1 [file metabolites-11-00006-s001.pdf]

Table S1. Logistic regression analysis for association between diabetic cardiovascular disease and five non-genetic factors transformed into categorical variables.

	DCVD	T2D only	Univariate†		Multivariate†	
**Characteristics***	**(*n* = 168)**	**(*n* = 2210)**	**OR (95% CI)**	***p***	**OR (95% CI)**	***p***
Men, N (%)	91 (54.2)	1159 (52.4)	1.07 (0.78–1.47)	0.666	1.18 (0.85–1.63)	0.337
Age, N (%)	-	-	-	-	-	-
<50 years	15 (8.9)	508 (23.0)	Reference	-	Reference	-
50–59	46 (27.4)	710 (32.1)	2.19 (1.21–3.97)	0.009	2.28 (1.25–4.18)	0.007
60≤	107 (63.7)	992 (44.9)	3.65 (2.11–6.34)	4.0×10^{-6}	3.75 (2.13–6.62)	5.1×10^{-6}
BMI, kg/m^2	-	-	-	-	-	-
<25	72 (42.9)	1088 (49.2)	Reference	-	Reference	-
25–29	80 (47.6)	947 (42.9)	1.28 (0.92–1.78)	0.147	1.31 (0.94–1.83)	0.112
30≤	15 (8.9)	160 (7.2)	1.42 (0.79–2.53)	0.240	1.67 (0.91–3.04)	0.096
SBP, mm Hg	-	-	-	-	-	-
<120	45 (26.8)	693 (31.4)	Reference	-	Reference	-
120–139	73 (43.5)	927 (41.9)	1.21 (0.83-1.78)	0.325	0.98 (0.66–1.45)	0.920
140≤	49 (29.2)	590 (26.7)	1.28 (0.84-1.95)	0.250	0.94 (0.61–1.45)	0.772
Creatinine, mg/dL§	-	-	-	-	-	-
<1.2 (1.0)	132 (78.6)	1957 (88.5)	Reference	-	Reference	-
1.2 (1.0)≤	36 (21.4)	253 (11.5)	2.11 (1.427–3.119)	1.8×10^{-4}	1.87 (1.25–2.81)	0.002

BMI, body mass index; CI, confidence interval; DCVD, diabetic cardiovascular disease; OR, odds ratio; SBP, systolic blood pressure; T2D, type 2 diabetes mellitus. * Data are shown as the number of subjects (percentage) for categorical variables. † ORs, 95% CIs, and *P* values were estimated by comparing 168 DCVD cases to 2210 T2D controls from the initial surveys of four cohort studies using both univariate and multivariate logistic regression analyses. ‡ Reference levels for serum creatinine were 1.2 and 1.0 mg/dL for men and women, respectively.

Table S2. Results of the linear mixed model analysis of 231 candidate SNPs for diabetic cardiovascular disease.

Gene	Chr	SNP	Function	N/R	LMM‡		
					RAF (Ca/Co)	OR	*P*
ZBTB40	1p36.12	rs12406194	3' UTR	G/A	0.07/0.03	1.08	2.0×10^{-4}
EPHB2	1p36.12	rs16827672	intron	A/G	0.65/0.55	1.03	3.9×10^{-4}
FOXE3	1p33	rs2820983	intergenic	A/G	0.70/0.60	1.03	6.0×10^{-4}
ATG4C	1p31.3	rs2803245	intron	T/C	0.39/0.30	1.03	8.5×10^{-4}
LOC101927560	1p31.1	rs6692233	intron	G/A	0.33/0.25	1.03	3.7×10^{-4}
TTLL7	1p31.1	rs685472	intron	C/A	0.43/0.33	1.03	3.0×10^{-4}
PRMT6	1p13.3	rs10494055	intergenic	T/C	0.05/0.02	1.09	4.3×10^{-4}
CELSR2, PSRC1	1p13.3	rs599839†	500bp~3′ UTR	A/G	0.09/0.06	1.04	0.015
LINC01768	1p13.3	rs6681769	intron	C/A	0.96/0.90	1.04	7.0×10^{-4}
MIR557	1q24.2	rs760686	intergenic	G/C	0.38/0.29	1.03	8.9×10^{-4}
MYOC	1q24.3	rs235858	intergenic	G/A	0.67/0.57	1.03	6.3×10^{-4}
KCNH1	1q32.2	rs1160574	intron	C/T	0.34/0.25	1.03	5.0×10^{-4}
ESRRG	1q41	rs4520477	intergenic	A/G	0.04/0.02	1.11	1.4×10^{-4}
KCNK1	1q42.2	rs10910201	intergenic	C/T	0.14/0.09	1.05	7.3×10^{-4}
FMN2	1q43	rs1341815	intron	T/G	0.44/0.34	1.03	3.2×10^{-4}
MYT1L	2p25.3	rs1364645	intergenic	G/A	0.38/0.30	1.03	6.9×10^{-4}
ALLC	2p25.3	rs17017879	intron	G/C	0.93/0.87	1.04	2.6×10^{-4}
ALK	2p23.2	rs4575680*	intron	G/C	0.08/0.04	1.07	9.0×10^{-5}
FAM176A	2p12	rs7581555	intergenic	T/C	0.79/0.71	1.03	7.3×10^{-4}
CTNNA2	2p12	rs6547315	intron	G/A	0.04/0.01	1.12	3.1×10^{-4}
LOC440900	2q14.1	rs202436	intergenic	A/G	0.18/0.12	1.04	7.0×10^{-4}
TFCP2L1	2q14.2	rs17006292†	intron	C/A	0.04/0.03	1.05	0.043
LRP1B, LINCO01853	2q22.1	rs1401939	intergenic	C/T	0.42/0.32	1.03	2.5×10^{-4}
LRP1B, LINCO01853	2q22.1	rs1527847	intergenic	T/C	0.30/0.21	1.03	4.1×10^{-4}
LRP1B, LINCO01853	2q22.1	rs1358410	intergenic	G/A	0.15/0.09	1.05	3.5×10^{-4}
ZEB2	2q22.3	rs1469346	intergenic	G/A	0.09/0.05	1.06	8.0×10^{-4}
KCNJ3	2q24.1	rs13410903	intergenic	A/G	0.19/0.12	1.04	5.5×10^{-4}
LOC105373718	2q24.2	rs6750818	intron (ncRNA)	C/A	0.14/0.08	1.05	9.7×10^{-4}
SP3	2q31.1	rs41326844*	intergenic	T/C	0.47/0.36	1.03	2.5×10^{-5}
PTH2R	2q34	rs1356376	intron	C/T	0.49/0.40	1.03	3.4×10^{-4}
MAP2	2q34	rs16842277	intergenic	A/T	0.74/0.63	1.03	1.5×10^{-4}
ABCA12	2q35	rs1004301	intergenic	C/T	0.04/0.02	1.11	2.5×10^{-4}
KCNE4	2q36.1	rs16864293*	intergenic	T/A	0.09/0.04	1.08	1.6×10^{-5}
ALPP	2q37.1	rs2741311	intergenic	C/T	0.04/0.01	1.11	6.8×10^{-4}
HDAC4	2q37.3	rs6706785†	intergenic	G/T	0.32/0.27	1.02	0.040
ZNF385D	3p24.3	rs9866313	intergenic	A/G	0.81/0.72	1.03	5.3×10^{-4}
GADL1	3p23	rs11920600	intron	G/A	0.05/0.03	1.08	9.1×10^{-4}
ERC2	3p14.3	rs2123184	intron	C/T	0.48/0.38	1.03	9.1×10^{-4}
SNTN	3p14.2	rs7626892	intron	T/C	0.64/0.54	1.03	7.8×10^{-4}
MAGI1	3p14.1	rs7430400	intron	C/T	0.75/0.66	1.03	4.9×10^{-4}
EPHA3	3p11.1	rs11920519	intron	A/G	0.31/0.22	1.03	4.7×10^{-4}
EPHA3	3p11.1	rs1512909	intron	C/T	0.09/0.04	1.07	2.1×10^{-4}
MIR548A3	3q13.11	rs16849422	intergenic	C/T	0.97/0.92	1.05	6.0×10^{-4}
LOC344595	3q13.12	rs9823214	intron	T/G	0.04/0.01	1.10	9.8×10^{-4}
LSAMP	3q13.32	rs4688064	intron	C/T	0.04/0.01	1.13	1.3×10^{-4}
SLC12A8	3q21.2	rs6789711	intron	A/C	0.04/0.02	1.11	1.1×10^{-4}
MRAS	3q22.3	rs9818870†	3' UTR	C/T	0.03/0.01	1.08	0.011
SPSB4	3q23	rs16851055†	intron (ncRNA)	G/A	0.23/0.18	1.02	0.036
C3orf79	3q25.2	rs16823176	intergenic	T/C	0.26/0.18	1.03	3.3×10^{-4}
C3orf79	3q25.2	rs924825	intergenic	T/C	0.71/0.61	1.03	8.2×10^{-4}
DLG1	3q29	rs3773844	intron	A/C	0.10/0.05	1.06	4.4×10^{-4}
PCDH7	4p15.1	rs10025805	intergenic	T/C	0.49/0.39	1.03	9.5×10^{-4}

GRXCR1	4p13	rs1369178	intergenic	G/T	0.74/0.64	1.03	2.8×10^{-4}
YIPF7	4p13	rs7698966	intergenic	A/G	0.62/0.52	1.03	5.8×10^{-4}
YIPF7	4p12	rs1439365	intergenic	C/T	0.47/0.44	1.02	8.5×10^{-4}
YIPF7	4p12	rs1595658	intergenic	T/C	0.42/0.48	1.03	3.8×10^{-4}
EPHA5	4q13.1	rs7692868	intergenic	A/G	0.03/0.01	1.12	9.3×10^{-4}
EPHA5	4q13.1	rs13150204	intergenic	G/A	0.03/0.01	1.13	5.5×10^{-4}
SULT1E1	4q13.3	rs991163	intergenic	A/G	0.26/0.19	1.03	7.3×10^{-4}
RUFY3	4q13.3	rs16845408	intron	G/A	0.11/0.06	1.05	7.3×10^{-4}
LOC100288146	4q24	rs17035270†	intron	C/T	0.99/0.04	1.04	0.028
LSM6	4q31.22	rs6816198	intergenic	C/T	0.30/0.22	1.03	5.7×10^{-4}
CTSO	4q32.1	rs10517631	intergenic	A/G	0.18/0.12	1.04	8.1×10^{-4}
GALNTL6	4q34.1	rs905571	intron	A/G	0.59/0.50	1.02	8.1×10^{-4}
FBXO8	4q34.1	rs10001837	intergenic	T/G	0.14/0.08	1.05	1.6×10^{-4}
SPCS3	4q34.3	rs1449738	intergenic	C/T	0.21/0.14	1.04	4.2×10^{-4}
VEGFC	4q34.3	rs6852384	intergenic	G/A	0.07/0.04	1.07	7.0×10^{-4}
MGC45800	4q34.3	rs17072597*	intron	C/T	0.22/0.14	1.05	1.5×10^{-4}
AHRR	5p15.33	rs6555242*	intron	T/G	0.07/0.03	1.09	3.1×10^{-4}
SLC9A3	5p15.33	rs1053226*	intron	C/T	0.05/0.02	1.11	1.8×10^{-5}
IRX1	5p15.33	rs2398625	intergenic	C/T	0.60/0.51	1.03	6.5×10^{-4}
LOC285696	5p15.1	rs297168	intron	G/C	0.11/0.06	1.05	5.5×10^{-4}
CDH6	5p13.3	rs10940845	intergenic	T/C	0.66/0.57	1.03	5.3×10^{-4}
GDNF	5p13.2	rs7445104	intergenic	G/A	0.19/0.12	1.04	3.5×10^{-4}
RGMB	5q15	rs17655764	intergenic	G/A	0.76/0.68	1.03	9.6×10^{-4}
EFNA5	5q21.3	rs152556	intron	T/A	0.30/0.22	1.03	7.3×10^{-4}
LOC107986441 (*KCNN2*)	5q22.2	rs4621553†	intron	A/G	0.09/0.05	1.05	0.002
PPIC	5q23.2	rs7723798	intron	G/C	0.13/0.07	1.05	9.6×10^{-4}
GRAMD3	5q23.2	rs12518322	intergenic	T/C	0.95/0.88	1.04	2.2×10^{-4}
GRAMD3	5q23.2	rs989374	intergenic	A/G	0.39/0.30	1.03	5.8×10^{-4}
SPOCK1	5q31.2	rs6893667*	intergenic	C/T	0.06/0.02	1.10	4.2×10^{-5}
SLIT3	5q35.1	rs17735410	intron	G/A	0.14/0.08	1.05	6.9×10^{-4}
SLIT3	5q35.1	rs6859754	intron	C/T	0.16/0.10	1.04	3.2×10^{-4}
HMP19	5q35.2	rs2913472*	intergenic	A/C	0.05/0.02	1.11	7.9×10^{-5}
FOXF2	6p25.3	rs932410	intergenic	G/T	0.80/0.71	1.03	3.9×10^{-4}
SCGN	6p22.2	rs1074707	intron	A/G	0.14/0.09	1.04	6.7×10^{-4}
ILRUN (*C6orf106*)	6p21.31	rs2814993†	intron	G/A	0.03/0.01	1.07	0.037
BTBD9	6p21.2	rs1883610	intron	C/T	0.05/0.02	1.09	9.7×10^{-4}
LRRC1	6p12.1	rs4455653	intron	C/A	0.44/0.46	1.02	9.3×10^{-4}
MYO6	6q14.1	rs2647410	intergenic	G/A	0.44/0.47	1.03	8.5×10^{-4}
IBTK	6q14.1	rs16893526†	intergenic	G/A	0.15/0.11	1.03	0.017
GPR6	6q21	rs9374133	intergenic	C/G	0.15/0.09	1.04	7.0×10^{-4}
MAN1A1	6q22.31	rs505838	intergenic	C/G	0.96/0.91	1.05	4.2×10^{-4}
RPS12	6q23.2	rs271173	intergenic	T/C	0.14/0.08	1.05	5.5×10^{-4}
STXBP5-AS1	6q24.3	rs652831	intron	G/A	0.47/0.43	1.03	5.0×10^{-4}
UST	6q25.1	rs2101154	intron	C/T	0.46/0.36	1.03	4.2×10^{-4}
ESR1	6q25.1	rs9371562	intron	A/T	0.96/0.90	1.04	8.9×10^{-4}
NOX3	6q25.3	rs9322547	intergenic	T/G	0.74/0.64	1.03	5.6×10^{-4}
NOX3	6q25.3	rs9371410	intergenic	C/T	0.30/0.22	1.03	2.9×10^{-4}
TULP4	6q25.3	rs341137	intron	T/G	0.09/0.04	1.07	2.9×10^{-4}
SLC22A2	6q25.3	rs677985	intron	T/C	0.19/0.13	1.04	9.0×10^{-4}
ISPD	7p21.2	rs7790551	intron	G/A	0.29/0.21	1.03	4.2×10^{-4}
ISPD	7p21.2	rs1918265	intron	T/C	0.49/0.38	1.03	1.9×10^{-4}
ISPD	7p21.2	rs6954901	intron	C/T	0.70/0.60	1.03	6.9×10^{-4}
RAPGEF5	7p15.3	rs1610046	intron	C/T	0.34/0.26	1.03	8.7×10^{-4}
CCDC129	7p14.3	rs1154846	intergenic	G/A	0.95/0.88	1.04	7.3×10^{-4}
POU6F2	7p14.1	rs7806341	intron	C/T	0.74/0.63	1.03	1.1×10^{-4}
YAE1D1	7p14.1	rs6947660	Lys68Glu	G/A	0.91/0.84	1.03	8.3×10^{-4}
AUTS2	7q11.22	rs6946893	intergenic	C/T	0.48/0.39	1.03	3.3×10^{-4}

MAGI2	7q21.11	rs1859459	intron	T/A	0.04/0.02	1.10	8.4×10^{-4}
PLXNA4	7q32.3	rs2880449	intron	C/T	0.14/0.09	1.04	8.0×10^{-4}
CNTNAP2	7q35	rs700279	intron	C/G	0.61/0.50	1.02	6.6×10^{-4}
CSMD1	8p23.2	rs10503223	intron	C/G	0.11/0.06	1.05	6.4×10^{-4}
MCPH1	8p23.2	rs4538911*	intergenic	C/G	0.13/0.06	1.08	5.0 × 10–7
LOC100287015	8p23.2	rs4454296	intergenic	A/G	0.11/0.06	1.06	1.8×10^{-4}
ST18	8q11.23	rs2450153*	intergenic	G/A	0.63/0.52	1.03	5.3×10^{-5}
ST18	8q11.23	rs3843918*	intergenic	T/C	0.46/0.44	1.03	7.0×10^{-5}
ZFPM2	8q23.1	rs10090372	intergenic	G/T	0.94/0.87	1.04	6.8×10^{-4}
TRPS1	8q23.3	rs2357045	intergenic	T/C	0.12/0.07	1.05	6.0×10^{-4}
LOC101927588	8q24.13	rs6470235	intron	C/A	0.63/0.53	1.02	9.3×10^{-4}
CCDC171	9p22.3	rs16933965	3' UTR	C/T	0.14/0.08	1.05	7.6×10^{-4}
MTAP	9p21.3	rs7865618†	intron	G/A	0.90/0.86	1.02	0.037
CDKN2B	9p21.3	rs1333042†	intron	A/G	0.71/0.65	1.02	0.025
LOC646700	9p21.1	rs10968749*	intergenic	A/G	0.19/0.12	1.04	7.4×10^{-5}
ACO1	9p21.1	rs13287216	intergenic	C/T	0.36/0.27	1.03	6.3×10^{-4}
APTX	9p21.1	rs1409692	intergenic	G/A	0.06/0.03	1.07	6.4×10^{-4}
TMEM2	9q21.13	rs4745106	intergenic	A/C	0.11/0.06	1.06	2.4×10^{-4}
PRUNE2	9q21.2	rs7847847	intron	C/T	0.33/0.25	1.03	2.1×10^{-4}
TLE4	9q21.31	rs1411160	intergenic	G/A	0.25/0.17	1.04	3.4×10^{-4}
TLE4	9q21.31	rs4877507	intergenic	G/T	0.34/0.25	1.03	1.8×10^{-4}
TLE4	9q21.31	rs11138359	intergenic	T/C	0.48/0.41	1.03	4.4×10^{-4}
SLC28A3	9q21.33	rs1077625	intergenic	A/G	0.10/0.05	1.05	9.9×10^{-4}
ZCCHC6	9q21.33	rs700768	intron	T/A	0.03/0.01	1.13	7.0×10^{-4}
LOC100507438	9q22.33	rs649057	intergenic	C/A	0.18/0.12	1.04	4.6×10^{-4}
TXNDC8	9q31.3	rs7035393	intergenic	T/C	0.07/0.04	1.07	7.5×10^{-4}
NRP1	10p11.22	rs767164*	intergenic	T/A	0.30/0.21	1.04	9.8×10^{-5}
GDF2	10q11.22	rs4922503	intergenic	G/C	0.83/0.75	1.03	6.7×10^{-4}
PCDH15	10q21.1	rs2589446	intron	T/C	0.43/0.34	1.03	2.4×10^{-4}
ZWINT, MIR3924	10q21.1	rs1503908*	intergenic	A/G	0.19/0.12	1.05	3.9×10^{-5}
EGR2	10q21.3	rs11813180	intergenic	T/C	0.85/0.77	1.03	4.5×10^{-4}
CTNNA3	10q21.3	rs10997139	intron	G/T	0.43/0.34	1.03	9.3×10^{-4}
SLC29A3	10q22.1	rs6480507	intergenic	T/C	0.49/0.41	1.03	2.9×10^{-4}
NRG3	10q23.1	rs4933830	intron	G/A	0.82/0.74	1.03	7.3×10^{-4}
LRIT2	10q23.1	rs11200928	Leu86=	G/A	0.41/0.32	1.03	8.1×10^{-4}
SORCS3	10q25.1	rs867626	intron	T/C	0.50/0.40	1.03	4.3×10^{-4}
TEAD1	11p15.3	rs12223927	intergenic	C/G	0.25/0.18	1.03	9.2×10^{-4}
CCDC34	11p14.1	rs11029951	intergenic	C/T	0.44/0.46	1.03	3.8×10^{-4}
CCDC34	11p14.1	rs7937671	intergenic	T/C	0.49/0.41	1.03	1.8×10^{-4}
PDHX	11p13	rs1346099	intergenic	G/C	0.13/0.07	1.05	7.5×10^{-4}
LDLRAD3	11p13	rs1001715*	intron	G/A	0.44/0.33	1.03	4.9×10^{-5}
LDLRAD3	11p13	rs12276510*	intron	G/A	0.43/0.33	1.03	5.5×10^{-5}
LDLRAD3	11p13	rs10082659	intron	G/T	0.46/0.36	1.03	2.8×10^{-4}
CD82	11p11.2	rs7946015*	intergenic	A/T	0.26/0.17	1.04	8.2×10^{-6}
TSPAN18	11p11.2	rs6485569	intergenic	C/A	0.36/0.27	1.03	3.6×10^{-4}
RNASEH2C	11q13.1	rs546202	intergenic	C/T	0.74/0.65	1.03	8.0×10^{-4}
PCNXL3	11q13.1	rs12801636†	intron	A/G	0.56/0.49	1.02	0.038
ODZ4	11q14.1	rs1945246	intron	T/G	0.11/0.06	1.05	6.7×10^{-4}
ODZ4	11q14.1	rs539136	intron	C/T	0.10/0.05	1.07	1.3×10^{-4}
ODZ4	11q14.1	rs507151	intron	G/A	0.03/0.01	1.12	6.2×10^{-4}
DLG2	11q14.1	rs668816	intron	C/T	0.47/0.38	1.03	9.4×10^{-4}
DLG2	11q14.1	rs349083*	intron	G/A	0.47/0.36	1.03	6.5×10^{-5}
NOX4	11q14.3	rs319025*	intron	T/C	0.67/0.56	1.03	4.1×10^{-5}
MIR1261	11q14.3	rs10501726*	intergenic	A/T	0.08/0.04	1.08	9.5×10^{-5}
MTNR1B	11q14.3	rs271030	intergenic	T/C	0.05/0.02	1.09	4.2×10^{-4}
ARHGAP20	11q23.1	rs10891220	intergenic	A/G	0.46/0.36	1.03	6.1×10^{-4}
LOC100507492	12p13.32	rs4474517	intergenic	G/A	0.40/0.32	1.03	9.2×10^{-4}

MREGP1	12p11.21	rs11610422†	intergenic	A/G	0.07/0.05	1.04	0.027
XPOT	12q14.2	rs11175379	intergenic	G/A	0.78/0.69	1.03	2.9×10^{-4}
MYF5	12q21.31	rs12231796	intergenic	A/T	0.78/0.69	1.03	3.6×10^{-4}
CRADD	12q22	rs11107184	intron	T/C	0.46/0.44	1.03	4.8×10^{-4}
CRADD	12q22	rs1872529	intron	G/C	0.42/0.32	1.03	1.9×10^{-4}
MYL2	12q24.11	rs3782889†	intron	G/A	0.88/0.83	1.02	0.046
TBX3	12q24.21	rs1895595	intergenic	A/G	0.22/0.15	1.03	7.5×10^{-4}
FGF9	13q12.11	rs9506827*	intergenic	T/C	0.29/0.20	1.04	5.9×10^{-5}
DCLK1	13q13.3	rs17053321	intron	C/A	0.10/0.05	1.06	5.6×10^{-4}
TRPC4	13q13.3	rs9576411	intergenic	C/T	0.41/0.49	1.02	9.3×10^{-4}
EPSTI1	13q14.11	rs2281813	3' UTR	A/G	0.68/0.58	1.03	2.9×10^{-4}
FAM124A	13q14.3	rs17837209	intron	G/A	0.35/0.27	1.03	8.3×10^{-4}
LINC00558	13q14.3	rs1458269	intron	T/C	0.69/0.59	1.03	6.2×10^{-4}
ATXN8OS	13q21.33	rs1341517	intergenic	A/G	0.31/0.21	1.03	1.3×10^{-4}
KLF12	13q22.1	rs9318232	intron	C/T	0.22/0.15	1.03	8.1×10^{-4}
CTAGE11P	13q22.2	rs529480	intergenic	A/G	0.45/0.35	1.03	1.3×10^{-4}
SLITRK6	13q31.1	rs17704575	intergenic	G/A	0.30/0.21	1.03	1.2×10^{-4}
SLITRK6	13q31.1	rs1538056	intergenic	G/A	0.49/0.39	1.03	1.3×10^{-4}
SLITRK6	13q31.1	rs1538050	intergenic	A/G	0.47/0.44	1.03	3.0×10^{-4}
LINC00410	13q31.3	rs17735224	intergenic	A/T	0.24/0.17	1.03	8.2×10^{-4}
METTL21EP, SLC10A2	13q33.1	rs9586032*	intergenic	G/A	0.23/0.15	1.04	7.4×10^{-5}
MYO16	13q33.3	rs9559436	intron	T/G	0.08/0.04	1.06	6.5×10^{-4}
FSCB	14q21.2	rs4906516	intergenic	G/A	0.10/0.05	1.06	1.4×10^{-4}
CEP128	14q31.1	rs8011469	intron	C/T	0.37/0.28	1.03	9.3×10^{-4}
C14orf64	14q32.2	rs877455*	intergenic	G/A	0.10/0.05	1.07	4.8×10^{-5}
BCL11B	14q32.2	rs1257290	intergenic	T/C	0.05/0.02	1.09	5.1×10^{-4}
MIRN656	14q32.31	rs8016145*	intergenic	G/A	0.09/0.04	1.08	6.4×10^{-5}
DIO3OS	14q32.31	rs6575819	intergenic	C/G	0.35/0.26	1.03	5.7×10^{-4}
RASGRP1	15q14	rs4924280	intergenic	G/C	0.44/0.47	1.03	7.5×10^{-4}
USP8	15q21.2	rs17431564	intergenic	C/T	0.20/0.13	1.04	7.0×10^{-4}
THSD4	15q23	rs8033309	intron	G/A	0.14/0.09	1.04	6.2×10^{-4}
MRPL46	15q25.3	rs16941706	intergenic	C/A	0.17/0.11	1.04	6.9×10^{-4}
SHISA9	16p13.12	rs8055417	intron	C/G	0.48/0.38	1.03	1.3×10^{-4}
KIAA0556	16p12.1	rs963999	intron	A/G	0.21/0.14	1.04	8.8×10^{-4}
CDH11	16q21	rs17465734*	intergenic	T/A	0.05/0.01	1.14	8.0×10^{-6}
ZFHX3	16q22.3	rs879324†	intron	A/G	0.67/0.62	1.02	0.022
DYNLRB2	16q23.2	rs11861602	intergenic	A/G	0.14/0.08	1.05	2.8×10^{-4}
DYNLRB2	16q23.2	rs10083792	intergenic	C/T	0.16/0.10	1.04	6.0×10^{-4}
JPH3	16q24.2	rs3860288	intron	C/T	0.75/0.65	1.03	3.5×10^{-4}
PITPNM3	17p13.2	rs11656015	intron	C/G	0.32/0.23	1.03	3.5×10^{-4}
PIRT	17p12	rs10852925	intergenic	T/C	0.67/0.57	1.03	6.8×10^{-4}
ELAC2	17p12	rs7212279	intergenic	G/C	0.18/0.12	1.04	4.0×10^{-4}
ELAC2	17p12	rs2674960	intergenic	A/G	0.40/0.30	1.03	2.3×10^{-4}
LOC100506974	17p12	rs4792405	intergenic	A/C	0.34/0.26	1.03	8.8×10^{-4}
CACNG5	17q24.2	rs8071471	intron	G/A	0.12/0.07	1.05	2.4×10^{-4}
L3MBTL4	18p11.31	rs694182	intron	T/C	0.43/0.46	1.03	2.0×10^{-4}
VAPA	18p11.22	rs16956185*	intergenic	G/A	0.15/0.08	1.06	3.2×10^{-5}
MC5R	18p11.21	rs1941092	intergenic	G/T	0.10/0.06	1.06	1.6×10^{-4}
KC6	18q12.3	rs2862365	intergenic	A/C	0.63/0.53	1.03	9.6×10^{-4}
CYP2B6	19q13.2	rs1872125*	intron	T/C	0.24/0.16	1.04	5.7×10^{-5}
CYP2B6	19q13.2	rs8192719	intron	C/T	0.21/0.14	1.04	2.2×10^{-4}
PLCB1	20p12.3	rs6055697	intron	C/A	0.60/0.50	1.03	3.4×10^{-4}
MAFB	20q12	rs1701853	intergenic	G/A	0.34/0.25	1.03	1.9×10^{-4}
LOC388813	21q11.2	rs462450	intron	C/T	0.43/0.34	1.03	6.5×10^{-4}
LOC100505973	21q21.1	rs2825255	intergenic	T/C	0.35/0.26	1.03	1.7×10^{-4}
PPIAL3	21q21.1	rs2825256*	intergenic	T/A	0.67/0.55	1.03	7.4×10^{-5}
LOC100505973	21q21.1	rs9982069*	intergenic	G/A	0.49/0.38	1.04	9.1×10^{-7}

LOC100505973	21q21.1	rs2205524	intergenic	T/A	0.68/0.58	1.03	3.0×10^{-4}
DGCR5	22q11.21	rs2075198	intron	A/G	0.22/0.14	1.04	1.9×10^{-4}
KLHL22	22q11.21	rs165807	intron	A/G	0.44/0.45	1.03	4.0×10^{-4}
CRYBB1	22q12.1	rs16982453	intergenic	G/A	0.11/0.06	1.05	4.1×10^{-4}
FAM19A5	22q13.31	rs2338258*	intergenic	T/C	0.13/0.07	1.06	3.6×10^{-5}
FLJ46257	22q13.31	rs9627183	intergenic	G/A	0.10/0.05	1.06	1.4×10^{-4}
FAM19A5	22q13.31	rs5768143*	intergenic	C/T	0.13/0.07	1.05	9.1×10^{-5}
FAM19A5	22q13.31	rs5768165*	intergenic	G/T	0.11/0.05	1.07	1.3×10^{-5}

Ca/Co, cases/controls; Chr., chromosome; LMM, linear mixed model; NA, not available; ncRNA, non-coding RNA; N/R, non-risk/risk allele; OR, odds ratio; RAF, risk allele frequency; SNP, single nucleotide polymorphism; UTR, untranslated region. [*] 32 SNPs associated with DCVD at $p < 10^{-4}$ in the LMM analysis. [†] 15 previously reported SNPs that were replicated in the current LMM analysis ($p < 0.05$) among 231 SNPs including 216 SNPs associated with DCVD at $p < 10^{-3}$. [‡] The risk allele frequencies (RAFs) were estimated for cases (left) and controls (right). ORs and *p*-values were estimated from LMM analysis after adjusting for age, sex, body mass index, and serum creatinine level.

Table S3. Incidence rates of cardiovascular disease in patients with type 2 diabetes and disease-free mortality rates in Korea.

Age Group	Person-Year[*]	Event, *N*[*]	Incidence Rate (95% CI)[*] (1000 person-year)	Disease-Free Mortality Rate (per 10,000 people)[†]
Both	-	-	-	-
40–45, years	1486	7	4.71 (2.25–9.88)	0.8
46–50	1090	14	12.84 (7.61–21.69)	1.1
51–55	932	13	13.95 (8.10–24.02)	1.7
56–60	1314	22	16.74 (11.02–25.43)	2.3
61–65	1390	26	18.71 (12.74–27.47)	3.4
66–69	1040	23	22.12 (14.70–33.28)	4.0
Total	7252	105	14.48 (11.96–17.53)	13.3
Men	-	-	-	-
40–45, years	944	3	3.18 (1.02–9.85)	1.1
46–50	748	10	13.37 (7.19–24.85)	1.6
51–55	534	5	9.36 (3.90–22.50)	2.4
56–60	672	11	16.37 (9.07–29.56)	3.4
61–65	578	15	25.95 (15.65–43.05)	5.0
66–69	458	9	19.65 (10.22–37.77)	5.9
Total	3934	53	13.47 (10.29–17.63)	19.5
Women	-	-	-	-
40–45, years	542	4	7.38 (2.77–19.66)	0.5
46–50	342	4	11.70 (4.39–31.16)	0.6
51–55	398	8	20.10 (10.05–40.19)	0.9
56–60	642	11	17.13 (9.49–30.94)	1.2
61–65	812	11	13.55 (7.50–24.46)	1.8
66–69	582	14	24.05 (14.25–40.62)	2.3
Total	3318	52	15.67 (11.94–20.57)	7.3

[*] Person-years, number of events, incidence rates, and 95% CIs were estimated with a 10-year follow-up data of the KARE Study. [†] Disease-free mortality data were obtained from the Korean Statistical Information System.

Table S4. Association results of risk prediction models for diabetic cardiovascular disease after stratification into risk quartiles in the case-control study.

Risk model*	Case/Control, N (%)	OR (95% CI)†	p†	Risk Model*	Case/Control, N (%)	OR (95% CI)†	P†
nGLT‡	167/2,195	AUC = 0.62 (0.57–0.66)		nGRS‡	167/2,195	AUC = 0.64 (0.60–0.68)	
Q1: 0.061–0.137	22 (13.2)/614 (28.0)	Reference	-	Q1: 0.000–0.976	27 (16.2)/7 38 (33.6)	Reference	
Q2: 0.149–0.204	50 (29.9)/669 (30.5)	2.09 (1.25–3.48)	0.005	Q2: 1.006–1.600	24 (14.4)/393 (17.9)	1.67 (0.95–2.93)	0.075
Q3: 0.205–0.251	35 (21.0)/464 (21.1)	2.11 (1.22–3.64)	0.008	Q3: 1.630–2.887	66 (39.5)/745 (33.9)	2.42 (1.53–3.83)	1.6×10^{-4}
Q4: 0.261–0.402	60 (35.9)/448 (20.4)	3.74 (2.26–6.18)	2.9×10^{-7}	Q4: 3.049–4.408	50 (29.9)/319 (14.5)	4.28 (2.63–6.97)	4.5×10^{-9}
GLT_{47}§	163/2,076	AUC = 0.71 (0.68–0.74)		PRS_{47}§	163/2076	AUC = 0.80 (0.77–0.83)	-
Q1: 0.119–0.164	4 (2.5)/556 (26.8)	Reference	-	Q1: 0.292–0.601	5 (3.1)/557 (26.8)	Reference	-
Q2: 0.164–0.186	17 (10.4)/543 (26.2)	4.35 (1.45–13.02)	0.009	Q2: 0.602–0.694	11 (6.8)/547 (26.4)	2.24 (0.77–6.49)	0.137
Q3: 0.186–0.255	71 (43.6)/489 (23.6)	20.2 (7.32–55.66)	6.4×10^{-9}	Q3: 0.695–0.800	27 (16.6)/533 (25.7)	5.64 (2.16–14.76)	4.2×10^{-4}
Q4: 0.255–0.417	71 (43.6)/488 (23.5)	20.2 (7.33–55.78)	6.3×10^{-9}	Q4: 0.801–1.496	120 (73.6)/439 (21.2)	30.5 (12.34–75.15)	1.2×10^{-13}
MLT_{47}	162/2,062	AUC = 0.73 (0.69–0.77)		MRS_{47}	162/2,062	AUC = 0.69 (0.65–0.73)	-
Q1: 0.042–0.120	14 (8.6)/542 (26.3)	Reference	-	Q1: 0.367–1.567	13 (8.0)/544 (26.4)	Reference	-
Q2: 0.120–0.176	19 (11.7)/537 (26.0)	1.37 (0.68–2.76)	0.379	Q2: 1.568–2.362	29 (17.9)/526 (25.5)	2.31 (1.19–4.49)	0.014
Q3: 0.176–0.221	27 (16.7)/529 (25.7)	1.98 (1.02–3.81)	0.042	Q3: 2.366–3.538	31 (19.1)/527 (25.6)	2.46 (1.27–4.76)	0.007
Q4: 0.221–0.384	102 (63.0)/454 (22.0)	8.70 (4.91–15.42)	1.3×10^{-13}	Q4: 3.539–5.430	89 (54.9)/465 (22.6)	8.01 (4.42–14.52)	7.2×10^{-12}

AUC, area under the receiver-operating curve; BMI, body mass index; CI, confidence interval; DCVD, diabetic cardiovascular disease; GLT, genetic liability threshold model; MLT, multifactorial liability threshold model; nGLT, non-genetic liability threshold model; OR, odds ratio; SNP, single nucleotide polymorphism. * Subjects were stratified into four risk quartiles based on the liabilities or risk scores contributed by genotyped variants, nongenetic factors, and combined effects of genetic and nongenetic factors (Q1, 0–25%; Q2, 25–50%; Q3, 50–75%; Q4, 75–100%). † ORs, 95% CIs, and P-values were estimated from logistic regression analysis in 168 DCVD cases and 2210 T2D controls. ‡ Both nGLT and nGRS models comprise four nongenetic factors: age, sex, BMI, and creatinine. § GLT_{47} and PRS_{47} are comprised of 47 SNPs, including SNPs identified at $p < 1 \times 10^{-4}$ and 15 reported SNPs that replicated in this study ($p < 0.05$).

Table S5. Predictability of genetic and nongenetic liability threshold models on diabetic cardiovascular disease in KARE, HEXA, Health2, and Twin-family Studies.

Study	-	KARE	HEXA	Health2	Twin-Family	Joint
Cases/Controls (N)	-	(68 / 1058)	(28 / 290)	(60 / 749)	(12 / 113)	(168 / 2210)
Risk Model*	No. of Risk Factor	AUC (95% CI)	AUC (95% CI)	AUC (95% CI)	AUC (95% CI)	AUC (95% CI)
nGLT	4	0.61 (0.55–0.67)	0.65 (0.53–0.77)	0.66 (0.59–0.72)	0.62 (0.44–0.79)	0.63 (0.59–0.67)
GLT_{47}	47	0.73 (0.68–0.78)	0.73 (0.64–0.81)	0.76 (0.70–0.81)	0.76 (0.58–0.93)	0.72 (0.69–0.76)
MLT_{47}	51	0.74 (0.68–0.80)	0.74 (0.63–0.85)	0.80 (0.74–0.86)	0.69 (0.52–0.87)	0.76 (0.71–0.79)
GLT_{231}	231	0.99 (0.99–1.00)	0.99 (0.99–1.00)	0.99 (0.99–1.00)	0.99 (0.97–1.00)	0.99 (0.96–1.00)
MLT_{231}	235	0.96 (0.94–0.99)	0.94 (0.88–1.00)	0.98 (0.97–1.00)	0.94 (0.88–1.00)	0.97 (0.95-0.98)

AUC, area under the receiver-operating curve; CI, confidence interval; GLT, genetic liability threshold model; MLT, multifactorial liability threshold model; nGLT, nongenetic liability threshold model; SNP, single nucleotide polymorphism.* GLT_{47} and GLT_{231} are comprised of 47 SNPs and 231 SNPs, including SNPs identified at $p < 1 \times 10^{-4}$ and $p < 1 \times 10^{-3}$, respectively, as well as 15 reported SNPs that replicated in this study ($p < 0.05$). The nGLT model comprises four nongenetic factors: age, sex, BMI, and creatinine.

Table S6. Predictability of genetic and nongenetic liability threshold models on diabetic cardiovascular disease after 10-fold cross validation test.

**Risk Model***	**nGLT**	**GLT**	**MLT**
Group	AUC (95% CI)†	AUC (95% CI)†	AUC (95% CI)†
47 SNPs	-	-	-
Group 1	0.72 (0.58–0.86)	0.71 (0.59–0.83)	0.85 (0.74–0.95)
Group 2	0.75 (0.64–0.85)	0.76 (0.64–0.87)	0.84 (0.75–0.94)
Group 3	0.63 (0.51–0.74)	0.74 (0.62–0.86)	0.72 (0.60–0.85)
Group 4	0.58 (0.45–0.71)	0.66 (0.57–0.76)	0.78 (0.65–0.91)
Group 5	0.57 (0.39–0.75)	0.68 (0.55–0.82)	0.67 (0.50–0.83)
Group 6	0.62 (0.50–0.73)	0.70 (0.59–0.81)	0.75 (0.64–0.86)
Group 7	0.66 (0.53–0.79)	0.74 (0.60–0.87)	0.74 (0.62–0.86)
Group 8	0.62 (0.50–0.74)	0.75 (0.67–0.83)	0.82 (0.72–0.92)
Group 9	0.54 (0.40–0.69)	0.76 (0.67–0.85)	0.65 (0.49–0.81)
Group 10	0.70 (0.55–0.84)	0.69 (0.59–0.80)	0.76 (0.60–0.91)
10-fold cross-validation	0.66 (0.62–0.70)	0.72 (0.68–0.75)	0.77 (0.73–0.81)
231 SNPs	-	-	-
Group 1	0.72 (0.58–0.86)	0.99 (0.98–1.00)	0.99 (0.98–1.00)
Group 2	0.75 (0.64–0.85)	0.98 (0.96–1.00)	0.98 (0.97–1.00)
Group 3	0.63 (0.51–0.74)	1.00 (1.00–1.00)	0.95 (0.91–1.00)
Group 4	0.58 (0.45–0.71)	1.00 (0.99–1.00)	0.96 (0.92–1.00)
Group 5	0.57 (0.39–0.75)	1.00 (0.99–1.00)	0.95 (0.89–1.00)
Group 6	0.62 (0.50–0.73)	1.00 (1.00–1.00)	0.98 (0.97–1.00)
Group 7	0.66 (0.53–0.79)	0.99 (0.98–1.00)	0.98 (0.95–1.00)
Group 8	0.62 (0.50–0.74)	0.99 (0.98–1.00)	0.99 (0.98–1.00)
Group 9	0.54 (0.40–0.69)	0.99 (0.99–1.00)	0.92 (0.84–1.00)
Group 10	0.70 (0.55–0.84)	0.99 (0.97–1.00)	0.93 (0.84–1.00)
10-fold cross-validation	0.66 (0.62–0.70)	0.99 (0.99–1.00)	0.98 (0.96–1.00)

AUC, area under the receiver-operating curve (ROC); CI, confidence interval; GLT, genetic liability threshold model; MLT, multifactorial liability threshold model; nGLT, nongenetic liability threshold model; SNP, single nucleotide polymorphism. * GLT_{47} and GLT_{231} are comprised of 47 SNPs and 231 SNPs, including SNPs identified at $p < 1 \times 10^{-4}$ and $p < 1 \times 10^{-3}$, respectively, as well as 15 reported SNPs that replicated in this study ($p < 0.05$). The nGLT model comprises four nongenetic factors, age, sex, BMI, and creatinine. † AUCs and 95% CIs were estimated by performing ROC analyses after 10-fold cross-validation tests.

Table S7. Reclassification improvement achieved by adding genetic markers to the multifactorial models for diabetic cardiovascular disease.

**Risk Model**	**Case/Control, N**	**NRI_e (95% CI)**	**NRI_{ne} (95% CI)**	**NRI (95% CI)***
Base model (Nongenetic model): nGLT or nGRS†				-
Enhanced model (Multifactorial model): Nglt + GLT or nGRS + PRS‡				-
GLT_{47}	168/2210	0.119 (0.006–0.245)	0.322 (0.261–0.380)	0.441 (0.280–0.606)
GLT_{231}	168/2210	0.905 (0.843–0.951)	0.919 (0.901–0.945)	1.824 (1752–1.883)
PRS_{47}	162/2062	0.444 (0.345–0.573)	0.572 (0.512–0.627)	1.017 (0.881–1.186)
PRS_{231}	113/1552	0.912 (0.837–0.967)	0.925 (0.898–0.956)	1.837 (1.747–1.911)

NRI, net reclassification index; NRI_e, event NRI; NRI_{ne}, non-event NRI; BMI, body mass index; CI, confidence interval; GLT, genetic liability threshold model; nGLT, non-genetic liability threshold model; nGRS, non-genetic risk score; PRS, polygenic risk score. * NRI values were estimated from 1000 bootstrap samples drawn without considering case/control status and predicted values were obtained through 10 fold cross-validation using a STATA command, incrsk. † Both nGLT and nGRS models comprise four nongenetic factors: age, sex, BMI, and creatinine. ‡ GLT and PRS consisted of 47-SNP ($p < 1 \times 10^{-4}$) and 231-SNP ($p < 1 \times 10^{-3}$) sets, respectively, including 15 reported SNPs that replicated in this study ($p < 0.05$).

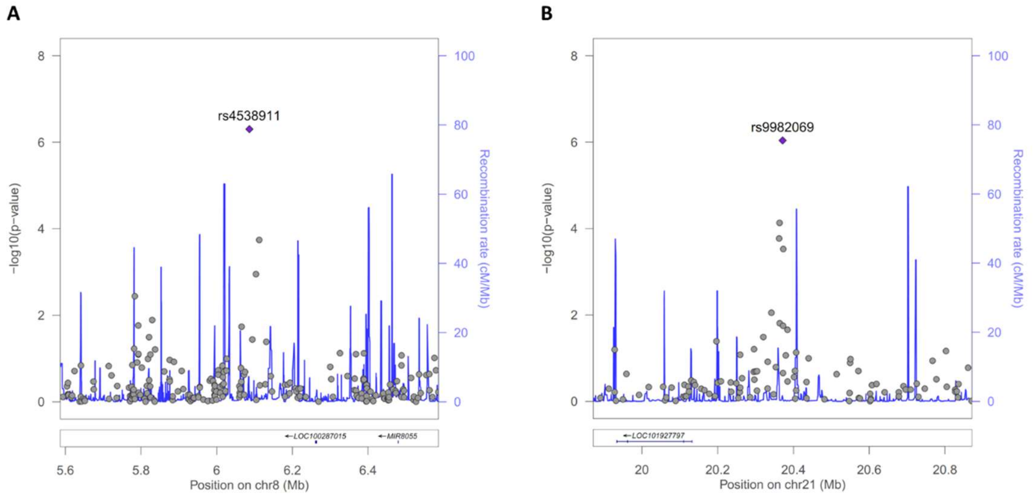

Figure S1. Regional association plots for regions containing each of two SNPs, rs4538911 (*LOC392180-MCPH1*, 8p23.2) and rs9982069 (*PPIAL3-SLC6A6P*, 21q21.1): X- and Y-axes indicate the chromosomal position (mega base, Mb) and -$\log_{10}$ transformed P value, respectively. The diamonds shown in purple indicate the most statistically significant SNPs in these regions, rs4538911 ($p = 5.0 \times 10^{-7}$) and rs9982069 ($p = 9.1 \times 10^{-7}$). The gray-filled circles indicate other SNPs within ± 500kb from the index SNP.

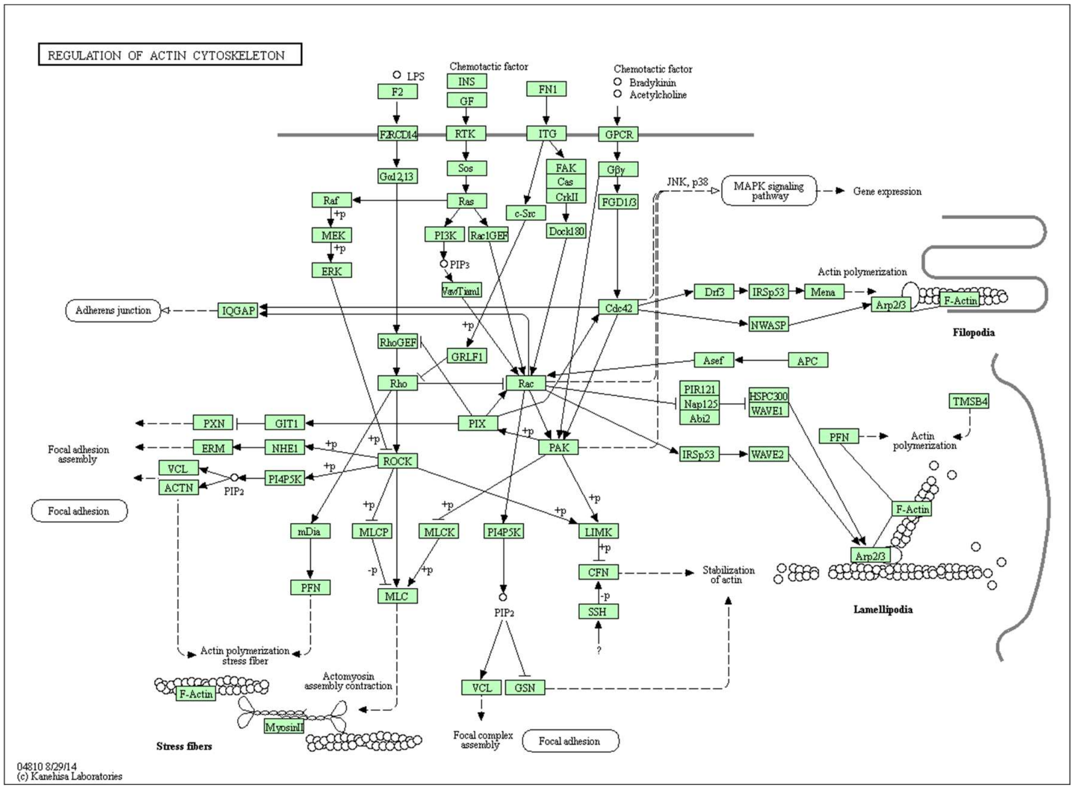

Figure S2. Schematic diagram of the regulation of actin cytoskeleton pathway (KEGG pathway, hsa04810): The gene set includes *MRAS* (3q22.3), *MYL2* (12q24.11), and *FGF9* (13p12.11) genes**.**

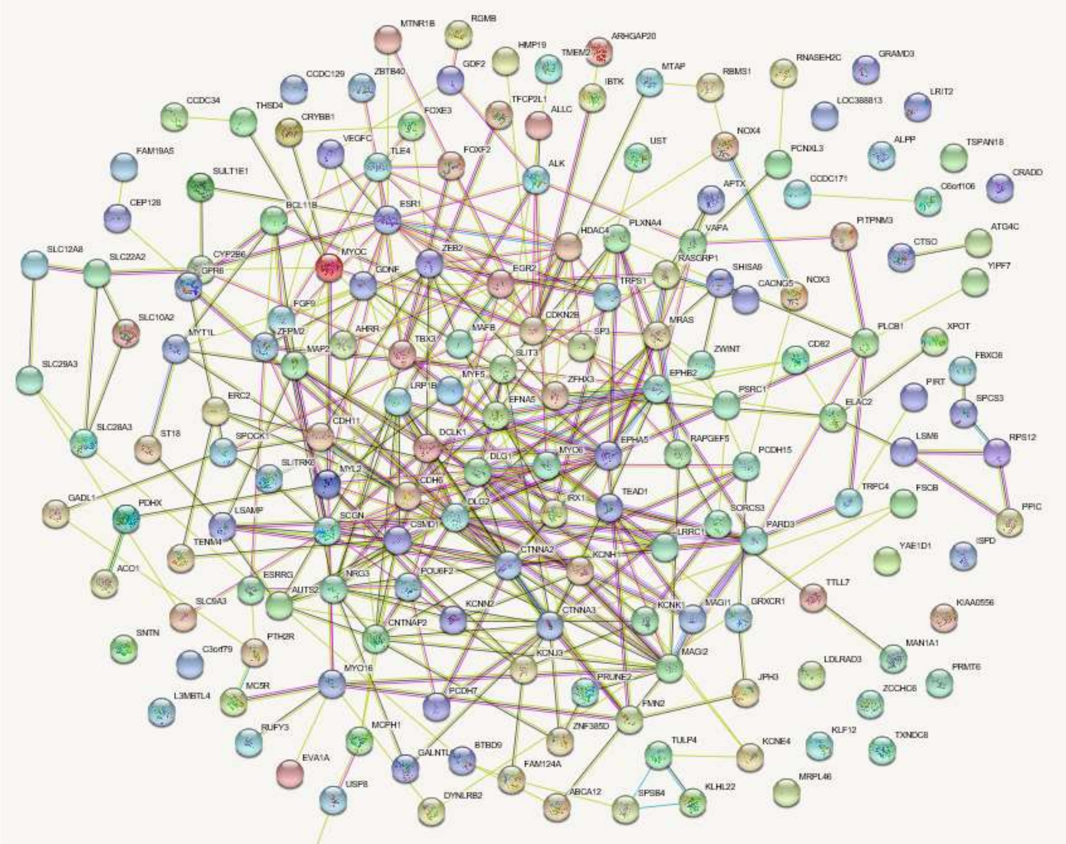

Figure S3. Protein-protein interaction network of 170 candidate genes for diabetic cardiovascular disease: Light-green line indicates the presence of copublications found through text mining; light purple, evidence of homology; purple line, experimental evidence of coexpression; black line, evidence of mRNA coexpression (confidence score of STRING, 0.25).

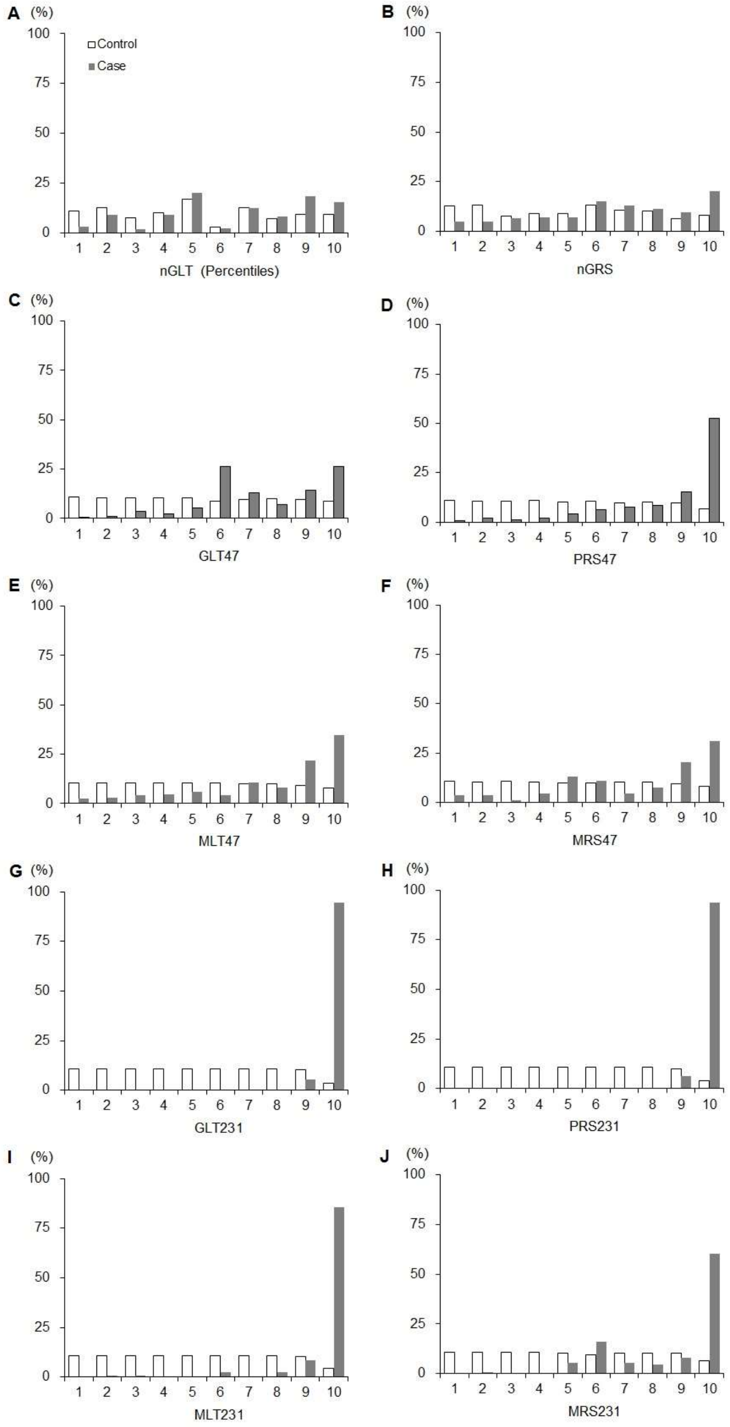

Figure S4. The frequencies of cases and controls and their risks of DCVD by risk percentiles for genetic (GLT), nongenetic (nGLT), and multifactorial liability threshold (MLT) models of 168 DCVD cases and 2,210 T2D controls: The non-genetic risk models, nGLT and nGRS, comprise four non-genetic risk factors, age, sex, body mass index, and creatinine (**A** and **B**). The genetic risk models comprise either 47-SNP sets (**C** and **D**) or 231-SNP sets (**G** and **H**). and The plots for the combined effects of genetic and nongenetic factors are shown for liability threshold models or weighted risk scores (White and gray bars denote the percentages of controls and cases, respectively (Y-axis). The X-axis display risk percentiles (1:10%, the lowest risk group −10:100%, highest risk group).
